# A Large‐Area Broadband Multimodal Dual‐Resonant Haptic Device for Bidirectional Telerobotic and Augmented Interactions

**DOI:** 10.1002/advs.75103

**Published:** 2026-04-07

**Authors:** Jihun Son, Joon Hyeok Kang, Jaeha Park, Yoon‐Gi Ku, Yongjun Lee, Jinhyung Kim, Minjin Kim, Jun Hyeok Lee, Junsik Shin, Minwoo Song, Gui Won Hwang, Dongbum Pyo, Gwanghyun Jo, Jeong‐Hoi Koo, Young‐Min Kim, Changhyun Pang, Tae‐Heon Yang

**Affiliations:** ^1^ School of Chemical Engineering Sungkyunkwan University (SKKU) Suwon Gyeonggi‐do Republic of Korea; ^2^ Department of Mechanical Engineering Konkuk University Seoul Republic of Korea; ^3^ Human‐Centric Robotics R&D Department Korea Institute of Industrial Technology Ansan‐si Gyeonggi‐do Republic of Korea; ^4^ Department of Mathematical Data Science Hanyang University ERICA Ansan‐si Gyeonggi‐do Republic of Korea; ^5^ Department of Mechanical and Manufacturing Engineering Miami University Oxford Ohio USA; ^6^ R&D Strategy Division Korea Institute of Oriental Medicine (KIOM) Daejeon Republic of Korea; ^7^ Samsung Advanced Institute for Health Sciences and Technology (SAIHST) Sungkyunkwan University Suwon Gyunggi‐do Republic of Korea

**Keywords:** bidirectional haptics, dual resonance, human–machine interface, large area

## Abstract

Bidirectional haptic systems demand interfaces that combine sensing and actuation, enabling concurrent detection of tactile inputs and delivery of perceptually rich feedback across a broad frequency spectrum. However, most existing haptic technologies remain limited to simple tactile sensing or narrowband feedback, struggling to resolve continuous motions and simultaneous sensory cues required for naturalistic human‐machine interaction (HMI). These limitations fundamentally constrain the fidelity and expressiveness of tactile communication, preventing current human–machine interfaces from reproducing the broadband of sensations perceived by human skin. Here, we present a large‐area bidirectional human–machine interface (HMI) that utilizes a unified haptic dual‐resonant actuator (UHDRA) capable of simultaneously implementing electrostatic‐based multimodal tactile sensing and actuation. The system provides spatially uniform tactile stimulation over a broad frequency range of 20–250 Hz while simultaneously enabling real‐time detection without interference from actuator‐induced vibration. Leveraging the actuator's intrinsic structural stiffness, the interface maintains stable vibration amplitudes into the gentle‐touch regime (≈2 N) while selectively modulating frequency, allowing clear discrimination of diverse tactile stimuli without perceptual discontinuity. Building on this decoupled bidirectional interaction capability, the proposed approach offers transformative potential for next‐generation applications such as bidirectional telerobotic and augmented interactions.

## Graphical abstract

We present a large‐area bidirectional human–machine interface integrating electrostatic multimodal tactile sensing with a dual‐resonant haptic actuator. The system enables linear pressure sensing that is decoupled from actuation and stable broadband vibrotactile feedback under dynamic contact conditions, supporting real‐time feedback modulation aligned with human perception. This scalable, power‐efficient platform advances telerobotic and augmented interaction.

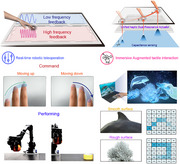

## 1 Introduction

The rapid expansion of artificial intelligence of things (AIoT) and metaverse technologies is redefining the fundamental nature of human‐machine interaction, accelerating the evolution of interfaces toward the coordinated integration of sensory perception and feedback [1, 2, 3, 4, 5]. In response to these demands, human‐machine interface (HMI) technologies have continued to evolve through parallel advances in the development of single and multimodal sensors that emulate the human tactile system, along with efforts to advance actuator technologies for delivering diverse and vivid tactile feedback [6, 7, 8, 9, 10]. These technological advances have accelerated the integration of sensing and actuation, enabling bidirectional systems in which real‐time sensory information is seamlessly translated into immediate and coordinated haptic responses. Despite these advances, achieving linear tactile sensing that remains robust under diverse operating conditions, along with spatially uniform tactile feedback across a broad frequency spectrum, remains a fundamental challenge.

Integrated bidirectional systems are widely employed across human‐machine interface (HMI) technologies applicable to real‐world environments through combinations of diverse sensors (e.g., strain sensitive [11, 12], piezoresistive [13, 14, 15], piezoelectric [16, 17, 18], triboelectric [19, 20]) and vibrotactile actuators (e.g., ERM [21, 22], piezoelectric [23, 24], DEA [25, 26]). However, sensors commonly used in current haptic interfaces rely on physical‐response mechanisms that are stable under static conditions but remain susceptible to ambient mechanical disturbances, making it difficult to maintain uniform and linear sensitivity under realistic operating conditions [27, 28]. In addition, actuators based on a single resonant architecture typically operate within a narrow frequency bandwidth (<30 Hz) that is often in the high‐frequency regime, fundamentally limiting the range and richness of tactile feedback achievable with a single actuator [29, 30]. These limitations hinder integrate system scalability, complicate dynamic sensing architectures such as motion sensing, and increase power consumption due to the use of multiple active elements.

To address these challenges, electrostatic‐based sensing mechanisms offer a promising alternative for tactile sensing in haptic interfaces. By detecting capacitance variations governed by minute changes in electrode separation under mechanical deformation, electrostatic self‐sensing provides a sensing response that is intrinsically decoupled from vibration‐induced dynamic actuation [31, 32, 33]. As a result, the electrical output reflects geometric deformation rather than frequency‐dependent material responses, enabling an immediate and linear response that remains robust under dynamic operating conditions without the need for separate sensing elements. Furthermore, electrostatic‐based actuators offer a promising solution for mitigating the reduction in tactile feedback caused by contact‐induced pressure in real‐world interaction environments [34, 35]. In contrast, actuators commonly used in conventional haptic interfaces exhibit load‐sensitive actuation behavior, leading to a pronounced attenuation of vibrotactile output under increasing contact forces [36, 37]. This limitation restricts consistent tactile feedback across varying contact conditions and fails to accurately reflect the mechanisms by which humans perceive touch under natural contact pressures.

Here, we present a large‐area bidirectional HMI that integrates electrostatic force based multimodal tactile sensing with a unified dual‐resonant haptic actuator (UHDRA) to cover the broadband of human tactile perception. By designing the spring stiffness to accommodate both low (<50 Hz) and high (>150 Hz) frequency regimes and integrating them into a large‐area unified actuator (150 mm × 90 mm), the UHDRA delivers vivid tactile stimulation across a broad frequency range. Notably, stable feedback performance is maintained under naturally occurring contact loads during diverse interactions with objects. Consequently, the integrated bidirectional HMI can resolve complex tactile signals containing multiple frequency components with high resolution, enabling the utilization of rich signal representations applicable to robotic teleoperation. In addition, real‐time pressure sensing allows dynamic modulation of feedback amplitude, enabling more immersive and spatially expressive tactile feedback in augmented tactile interaction and offering transformative potential for next‐generation advanced human‐machine interfaces.

## 2 Results and Discussion

### 2.1 Conceptual Illustration of a Bidirectional HMI and Characteristics of UHDRA

**FIGURE 1:**
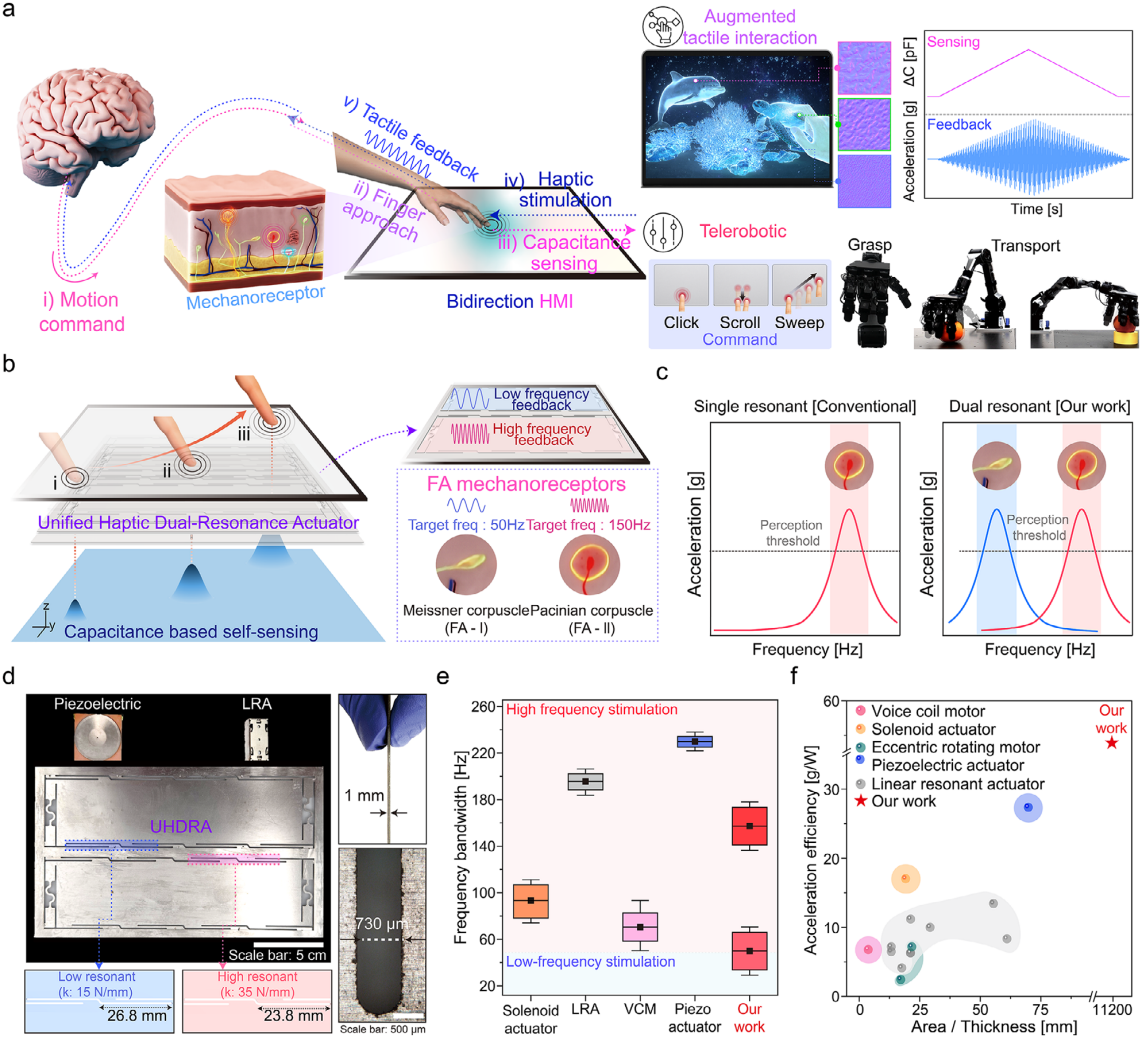
Concept of a bidirectional HMI and characteristics of UHDRA. (a) Schematic of the neural tactile sensing system and its operating principle, including applications of the integrated bidirectional HMI. (b) Conceptual illustration of electrostatic sensing and dual‐resonant actuation enabling continuous dynamic touch feedback, inspired by FA mechanoreceptor frequency responses. (c) Comparison between conventional single‐resonant actuators and the dual‐resonant UHDRA, highlighting the expanded vibrotactile frequency range enabled by the dual‐resonant architecture. (d) Photograph of representative haptic actuators and schematic illustration of the UHDRA, showing the geometric design of the dual‐resonant spring architecture, including spring constant, rib length and overall thickness. (e) Comparison of vibrotactile frequency bandwidth between the UHDRA and conventional haptic actuators. (f) Acceleration efficiency normalized by the area‐to‐thickness ratio for the UHDRA compared with conventional haptic actuators.

Figure 1a presents the overall concept of a bidirectional human‐machine interface (HMI) together with the design principles and performance characteristics of the unified haptic dual‐resonant actuator (UHDRA). Electrostatic‐based capacitive self‐sensing and vibrotactile feedback are integrated on a shared surface, allowing tactile input and output to coexist within a single interface. Contact pressure naturally applied by the user induces changes in electrode separation, which are detected in real time as capacitance variations and immediately translated into corresponding vibrotactile responses through the same platform (Figures S1 and S2). This bidirectional interaction framework precisely reflects user intent while ensuring large‐area uniformity, thereby enabling both physical interaction for robotic control and intuitive, realistic tactile experiences in augmented reality environments. At the core of this bidirectional interface, structural deformation is directly transduced through capacitive self‐sensing, enabling continuous and linear tactile input detection without the need for discrete electrode arrays (Figure 1b). This intrinsic sensing capability supports large‐area motion recognition, including contact translation and continuous touch dynamics. Simultaneously, the actuator incorporates two mechanically distinct resonant modes designed to selectively stimulate FA‐I (Meissner corpuscles) and FA‐II (Pacinian corpuscles), thereby providing perceptually rich tactile feedback across the frequency range relevant to human touch perception [38]. Owing to this dual‐resonant architecture, the frequency response of the UHDRA differs fundamentally from that of conventional single‐resonant actuators. Whereas single‐resonant designs generate perceptible stimulation only within a narrow frequency window near a single resonance peak, the UHDRA sustains effective tactile output in both low‐ and high‐frequency regimes, substantially broadening the usable stimulation bandwidth and more comprehensively covering the human tactile perceptual spectrum (Figure 1c). The dual‐resonant behavior is enabled by structural stiffness engineering in a large‐area (150 mm × 90 mm), lightweight (≈115 g), ultra‐thin (<1 mm) architecture. Modulation of spring length allows spatial separation of low‐ (≈15 N mm^−1^) and high‐resonance (≈35 N mm^−1^) regions, as verified by optical and cross‐sectional images (Figure 1d, Figures S3–S6). Owing to this structural design, the UHDRA offers a substantially broader tactile stimulation range, achieving an effective bandwidth of ≈80 Hz across low‐ and high‐frequency regions, compared with the ≈40 Hz, high‐frequency‐biased bandwidth of conventional actuators (Figure 1e and Table S1) [39, 40, 41, 42, 43, 44]. Moreover, when vibration acceleration efficiency is normalized by actuator area, thickness, and power consumption, the UHDRA delivers high acceleration output at the lowest power consumption while maintaining the largest area and thinnest profile among the compared actuators (Figure 1f, Figures S7 and S8 and Table S2) [23, 45, 46, 47, 48, 49, 50, 51, 52, 53]. Together, these results establish the UHDRA as a scalable and energy‐efficient solution for large‐area haptic interfaces capable of delivering perceptually rich tactile feedback.

### 2.2 Structural Design and Dual‐Resonant Vibrotactile Performance of the UHDRA

**FIGURE 2:**
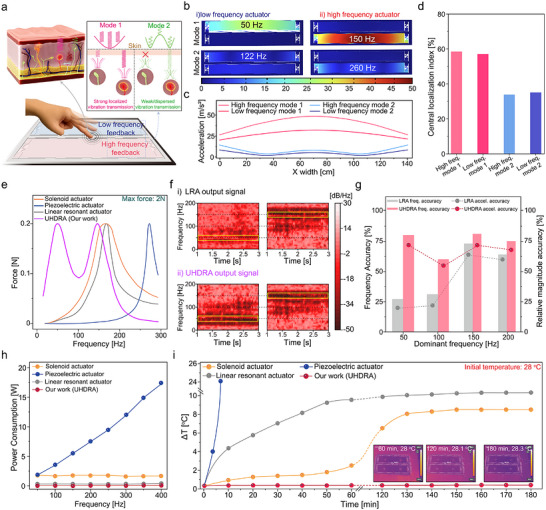
Design and performance of UHDRA. (a) Conceptual schematic illustrating mode‐dependent vibration transmission in the UHDRA. (b) FEM‐based modal simulations of two actuators. (c) Acceleration profiles along the horizontal direction of the actuator for two resonant modes. (d) Central localization index for each resonant mode, quantifying vibration concentration in the actuator center. (e) Frequency‐acceleration responses of the UHDRA compared with haptic actuators under a maximum output force of 2 N (solenoid, piezoelectric, and linear resonant actuators). (f) STFT spectrograms of output acceleration signals from the LRA and UHDRA in two distinct frequency regions. (g) Frequency and acceleration reproduction accuracy of the LRA and UHDRA for aperiodic inputs with dominant frequencies from 50 to 200 Hz. (h) Comparison of power consumption between the UHDRA and actuators at each resonant frequency under comparable output conditions. (i) Time‐dependent mechanical degradation of each actuator under continuous operation.

Figure 2a schematically illustrates the mode‐dependent vibration transmission characteristics realized within a single UHDRA. From the perspective of tactile perception, mode 1, in which vibrational energy is concentrated in a localized region, is known to provide clear and well‐defined tactile cues at the contact point [54]. Accordingly, we performed finite‐element‐based analyses (FEM) in which spring length and stiffness were systematically tuned as key design parameters to theoretically identify conditions that induce mode 1 behavior in both the low‐frequency (≈50 Hz) and high‐frequency (≈150 Hz) regimes (Figure 2b). When operated in mode 1, the UHDRA generates a localized and intensified vibration near the actuator center, which propagates toward the periphery while maintaining relatively high acceleration levels across the structure. In contrast, mode 2 exhibits a more spatially distributed vibration profile with overall lower acceleration, making it less effective for delivering sharp tactile cues (Figure S8). Additional displacement analysis confirms that the peak displacement in mode 1 is more than two times larger than that in mode 2 (Figure S9 and Movie S1). These trends are further corroborated by experimentally measured acceleration distributions along the horizontal direction of the actuator (Figure 2c). Compared with mode 2, mode 1 maintains higher acceleration levels across the entire actuator area, with particularly pronounced differences near the center. To quantitatively evaluate the spatial distribution of vibrational energy, a central localization index was introduced. The results show that mode 1 maintains vibration propagation across the structure while exhibiting stronger energy concentration near the center, whereas mode 2 preserves a comparable propagation range but with weaker and more uniformly distributed vibrational energy (Figure 2d and Note S1). Beyond spatial characteristics, the UHDRA demonstrates superior broadband vibrotactile performance compared with conventional haptic actuators. As shown in Figure 2e, under a maximum output force of 2 N, the UHDRA exhibits pronounced acceleration responses in both low‐ and high‐frequency regions, whereas solenoid, piezoelectric, and linear resonant actuators show effective responses only within a narrow frequency band near a single resonance (Figures S10 and S11 and Notes S2 and S3). This capability enables a single actuator to effectively deliver diverse tactile frequency components. The dynamic response characteristics were further evaluated by analyzing the rising and falling times. Among the compared actuation mechanisms, the electrostatically driven UHDRA exhibits the fastest response in both metrics (rising time: 8 ms; falling time: 26 ms), indicating superior temporal responsiveness (Figures S12–S14 and Notes S4 and S5). Dynamic signal reproduction fidelity was assessed through short‐time Fourier transform (STFT) analysis in comparison with a linear resonant actuator (LRA) (Figure 2f and Figure S12). When driven outside its resonance, the LRA exhibits pronounced frequency distortion and mode‐dominant behavior, whereas the UHDRA accurately reproduces target frequencies in both low‐ and high‐frequency regimes. This distinction is quantitatively confirmed by frequency and acceleration reproduction accuracy analysis for aperiodic inputs spanning 50–250 Hz (Figure 2g). Across this range, the UHDRA consistently outperforms the LRA, with particularly pronounced advantages in the low‐frequency region (50–100 Hz), where it maintains frequency accuracies of approximately 60–80% and acceleration accuracies of approximately 55–70%, demonstrating the effectiveness of the dual‐resonant architecture for broadband dynamic signal reproduction. In addition to performance fidelity, the UHDRA exhibits favorable energy efficiency and mechanical robustness. As shown in Figure 2h, the power consumption of the piezoelectric actuator increases nearly linearly as the driving frequency approaches 400 Hz, reaching the tens‐of‐watts range, whereas the solenoid actuator and the LRA remain at the few‐watts level. In contrast, the electrostatically driven UHDRA maintains power consumption below 1 W across the tested frequency range and below approximately 0.4 W in most cases (Figure S15). This low electrical load is directly reflected in thermal measurements. During 180 min of continuous operation, the piezoelectric actuator exhibits a temperature rise exceeding 24°C within only 7 min of operation, while the linear resonant actuator (LRA) and solenoid actuator show temperature increases ranging from several degrees to nearly 10°C. By comparison, the UHDRA exhibits negligible temperature change under identical conditions (Figure 2i and Movie S2). Collectively, these results demonstrate that the structural design of the UHDRA enables spatially controllable vibration, broadband frequency response, high‐fidelity dynamic signal reproduction, and long‐term operational stability, establishing the UHDRA as a promising platform for large‐area, low‐power, and high‐reliability haptic interfaces.

### 2.3 Integrated Bidirectional HMI and Characteristics of Sensing Linearity and Feedback Uniformity

**FIGURE 3:**
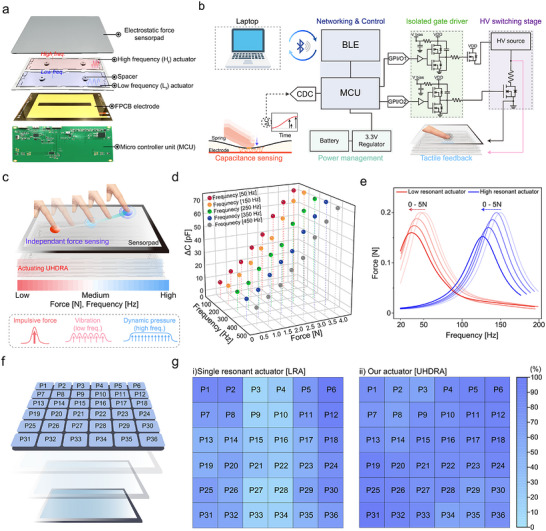
Schematics of the integrated bidirectional HMI and its decoupled sensing linearity and feedback uniformity. (a) Exploded view of an integrated bidirectional HMI comprising five independently controlled units for multimodal haptic feedback. (b) Block diagram of signal processing from sensing to vibrotactile feedback. (c) Schematic of decoupled force sensing and vibrotactile actuation, enabling vibration‐independent linear pressure sensing, across the human perceptible tactile range. (d) Capacitance change (ΔC) under simultaneous vibration and applied pressure, demonstrating linear, decoupled pressure sensing during active vibrotactile stimulation. (e) Performance of the UHDRA in maintaining stable vibrotactile acceleration across 0–5 N. (f) Schematic of 36 sensing regions (6 × 6 array) illustrating spatial uniformity of vibrotactile. (g) Comparison of spatial uniformity in vibration transmittance under pressure induced condition between the LRA and the UHDRA across 36 regions.

Figure 3a presents the vertically expanded architecture of the proposed bidirectional human‐machine interface (HMI), in which electrostatic‐based capacitive self‐sensing and vibrotactile actuation are integrated on a single surface. The system comprises a touchpad with embedded electrostatic sensors, spatially separated actuator layers for low‐ and high‐frequency operation, an FPCB electrode layer, and an underlying microcontroller unit (MCU), collectively enabling the seamless integration of sensing and actuation within a large‐area yet compact form factor. The corresponding signal‐processing flow is illustrated in Figure 3b. When touch or pressure is applied by the user, capacitance variations induced by changes in electrode separation are converted into digital signals via a capacitance‐to‐digital converter (CDC) and transmitted to the MCU. Based on this sensing information, the MCU generates real‐time control signals that drive the high‐voltage switching stage through an isolated gate driver, thereby producing vibrotactile feedback. This architecture intrinsically couples sensing and actuation, enabling fully bidirectional operation without additional sensing layers (Figure S16 and Note S7). A key advantage of the electrostatic self‐sensing mechanism is its ability to maintain linear pressure sensing even under simultaneous vibrotactile actuation across the fast‐adapting (FA) afferent range of human‐perceptible tactile frequencies. This robustness arises because the sensing signal directly reflects instantaneous structural deformation specifically, changes in electrode separation rather than frequency‐dependent material property variations. As a result, accurate and reliable tactile input recognition is preserved under dynamic vibration conditions. In parallel, the UHDRA maintains high‐fidelity vibrotactile output even when structural deformation is induced by external loads (Figure 3c). To validate these characteristics, capacitance changes (Δ*C*) were analyzed under combined pressure (≈ 4 N) and vibration (≈ 450 Hz) loading conditions (Figure 3d and Figure S17). Across all tested conditions, Δ*C* exhibited a monotonic and clearly distinguishable response, confirming that linear sensing performance is stably maintained during actuation. Furthermore, considering the loading conditions typically applied when tactile sensations are perceived during physical interaction, we analyzed the vibration acceleration under different applied loads. As shown in Figure 3e, within the applied load range of 0–2 N, the vibration acceleration remained nearly constant despite the natural shift in the resonance frequency, satisfying the conditions required for stable tactile feedback during interaction with real objects (Figure S17). However, when a larger load of approximately 5 N was applied, a reduction in vibration acceleration of about 20% and 25% was observed in the respective actuator regions. Nevertheless, these values remain sufficient to provide perceivable vibration feedback on the skin surface. In addition to the load‐insensitive temporal stability maintained within the applied load range up to 2 N, the spatial uniformity of vibrotactile feedback under tactile interaction forces was also evaluated. To evaluate spatial uniformity, the vibration distribution was analyzed across the entire 6 × 6 grid composed of 36 sensing regions (Figure 3f). For comparison, an array of six linear resonant actuators (LRAs) arranged in a 2 × 3 configuration was fabricated over the same device area as the UHDRA, and the same uniformity test was performed. In addition to evaluating the response at individual positions, eight analysis paths were defined across the 36‐grid area to assess the spatial connectivity and consistency of vibration propagation along different directions (Figure 3g and Figure S18). In the LRA array, the vibration amplitude exhibits large spatial variations due to pressure‐induced shifts in resonance frequency, device‐to‐device variability, and non‐uniform boundary conditions. These results reflect inherent limitations of multi‐actuator array architectures, including reduced spatial resolution and modal interference. In contrast, the UHDRA transmits vibrations generated by the actuator to the sensor pad surface through four spacers arranged in a 2 × 2 configuration around the actuator. Owing to its continuous and distributed mechanical structure, the device maintains a uniform vibration distribution across the entire surface, enabling consistent tactile feedback regardless of the contact location.

### 2.4 Demonstration of Real‐Time Robotic Teleoperation

**FIGURE 4:**
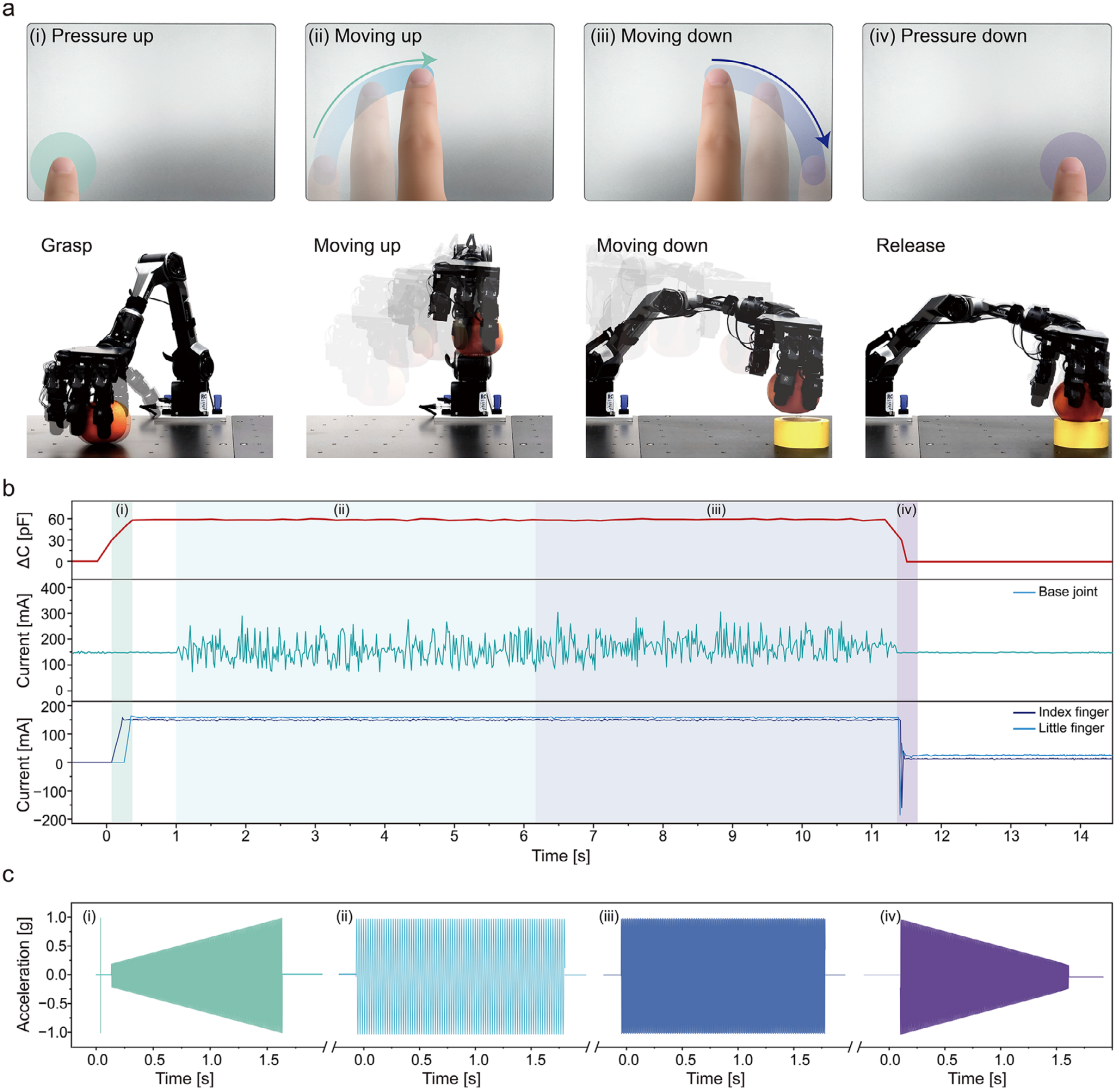
Demonstration of real‐time bidirectional robotic teleoperation. a) Operation sequence of a command‐driven teleoperated robotic hand, illustrating grasping, lifting, positioning, and releasing actions, together with representative multimodal tactile gesture inputs used as intuitive command signals: (i) pressure increase to close the hand, (ii) upward finger motion to command upward arm movement, (iii) downward finger motion to command downward arm movement, and (iv) pressure release to open the hand. (b) Time‐synchronized measurements showing the capacitance change (ΔC), the current of the robot arm base joint, and the currents of the index and little finger motors during the four sequential gesture phases. (c) Representative feedback signals from the UHDRA delivered to the user fingertip.

To validate the application potential of the real‐time bidirectional teleoperation platform implemented with the integrated system, we realized a user‐robot hand interaction framework that combines capacitive pressure sensing with vibrotactile feedback. As shown in Figure 4a, intuitive multimodal tactile gestures applied to the UHDRA surface are mapped to continuous robotic motion commands, allowing the user to naturally perform grasping, lifting, positioning, and releasing actions. Specifically, an increase in applied pressure commands hand closing, upward and downward finger motions correspond to upward and downward movements of the robotic arm, respectively, and pressure release triggers hand opening. This gesture design enables intuitive command input based solely on tactile interaction, without the need for additional input devices or visual interfaces (Figure S19 and Note S11). The corresponding sensing and robotic actuation responses to these input gestures are shown in Figure 4b. Capacitance changes (Δ*C*) measured on the touchpad surface clearly distinguish the four gesture phases, while the base joint current of the robotic arm and the finger motor currents evolve in temporal synchrony with the issued commands. During the grasping phase, increased finger motor currents reflect object contact and grasping, whereas during arm motion phases, variations in the base joint current indicate upward and downward arm movements. In contrast, the release phase is characterized by a rapid decrease in both capacitance and motor currents, clearly delineating hand opening and object release. These results demonstrate that capacitive self‐sensing signals can serve as reliable real‐time inputs for robotic control. Simultaneously with command execution, the UHDRA delivers vibrotactile feedback to the user's fingertip, thereby completing bidirectional control (Figure 4c). Among the twelve static and dynamic multimodal tactile feedback patterns available in the system, four representative patterns were selected for the teleoperation scenario (Figure S20 and Note S12). Grasp‐related events are encoded as vibration patterns with a gradually increasing amplitude envelope, reflecting the increasing motor load during object interaction. In contrast, vertical arm motions are conveyed using frequency‐selective vibration patterns that exploit the dual‐resonant characteristics of the UHDRA, with upward movements mapped to the high‐frequency resonant mode and downward movements mapped to the low‐frequency resonant mode (Movie S3). Together, these demonstrations show that the integrated sensing and dual‐resonant actuation architecture of the UHDRA enables real‐time discrimination of distinct feedback signals through combined amplitude and frequency modulation, providing high‐fidelity tactile feedback that reflects the physical state of interaction and effectively supports real‐time bidirectional interaction in teleoperation environments.

### 2.5 Demonstration of Real‐Time Augmented Tactile Interaction with Spatially Expressive Tactile Feedback

**FIGURE 5:**
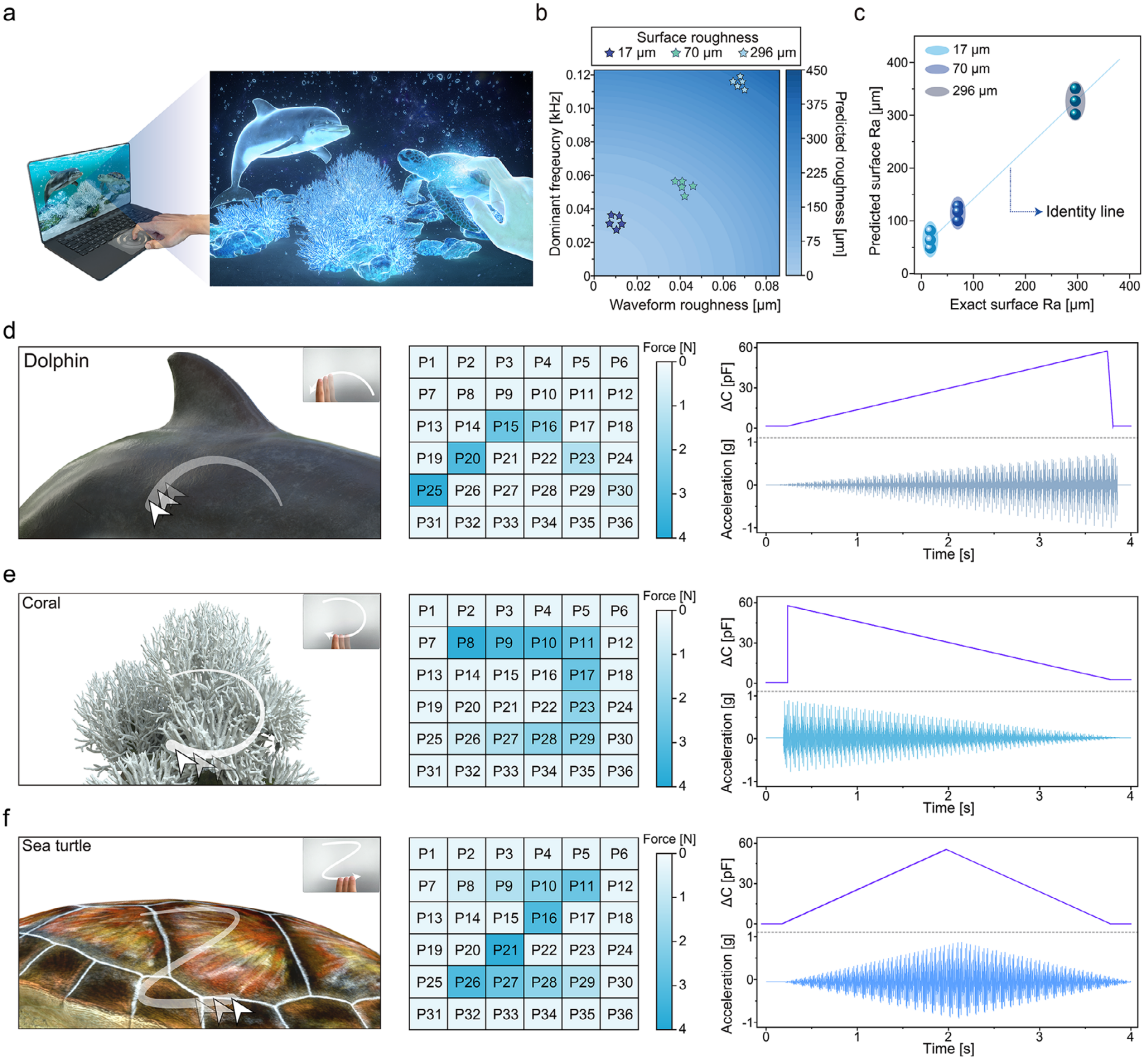
Demonstration of real‐time augmented tactile interaction with spatially expressive tactile feedback. (a) Conceptual illustration of a UHDRA integrated touch interface in which touch position and applied pressure are sensed and mapped to texture related vibrotactile signals during interaction with visually rendered marine objects. b) Contour map showing the empirical relationship between dominant vibration frequency, waveform roughness index, and estimated surface roughness. (c) Correlation between reference surface roughness values and roughness values predicted from vibration features, based on the sandpaper calibration dataset. (d–f) Vibrotactile renderings emulating three representative surface textures: dolphin skin (smooth), coral (intermediate roughness), and turtle shell (rough) with amplitude varying under continuous motion and applied pressure.

Figure 5a illustrates the concept of an augmented tactile interaction that combines visually rendered marine objects typically difficult to experience directly under realistic human interaction conditions with corresponding vibrotactile signals. In this demonstration, surface roughness characteristics associated with dolphin skin, coral, and turtle shell are modeled and translated into vibration profiles generated by the UHDRA, enabling tactile reproduction of these textures (Figure S21). Importantly, the vibrotactile signals are not mapped based on subjective texture perception but instead are derived from features directly linked to physical surface roughness parameters. To establish a quantitative relationship between the actuator's vibration output and surface roughness, two key features were extracted from the measured acceleration signals: a waveform roughness index and the dominant frequency (Figure 5b, Figure S22, and Note S13). This feature extraction approach follows established surface roughness evaluation methods. A calibration dataset was constructed using sandpaper samples spanning a wide range of surface roughness values, and identical vibration measurement and feature extraction procedures were applied to each sample. Using reference samples with known arithmetic mean roughness (*R*a), an empirical mapping was derived between the feature space and Ra values (Figure 5c and Note S14). The resulting predicted roughness values exhibit a consistent correlation with the reference *R*a values, indicating that the extracted vibration features reliably capture physical surface roughness. Figure 5d–f present the implementation of texture‐based vibrotactile rendering on the UHDRA touch interface using this mapping framework. As a user moves a finger across the displayed object, the system continuously acquires both the contact position and the applied pressure. The capacitance‐based pressure signals are spatially resolved by dividing the touch surface into a 6 × 6 grid, enabling position‐resolved pressure sensing. For each grid cell, the amplitude of the generated vibrotactile output is scaled proportionally to the locally measured pressure, such that increased contact force results in increased vibration amplitude. In contrast, the frequency and waveform characteristics associated with surface roughness remain unchanged, allowing pressure and texture information to be independently encoded. The resulting vibration profiles are rendered continuously along the finger trajectory, with their amplitudes modulated in real time according to the applied pressure. Consequently, smooth surfaces are represented by low‐amplitude, stable vibrations, whereas rough surfaces produce stronger and more irregular vibration patterns, yielding a tactile experience consistent with the corresponding visual textures (Figure S23 and Note S15). Taken together, these demonstrations show that the UHDRA can simultaneously support texture‐based vibrotactile signals and interaction‐oriented tactile feedback on a single large‐area touch surface (Movie S4). This capability highlights the potential of the UHDRA as a tactile interface for augmented reality environments, enabling real‐time delivery of vibrotactile feedback that is spatially and physically aligned with visual information.

## 3 Conclusion

We have presented a large‐area bidirectional human–machine interface that integrates electrostatic multimodal tactile sensing with a unified dual‐resonant haptic actuator, addressing key challenges in linear tactile sensing and broadband vibrotactile feedback within a single platform. The capacitance‐based self‐sensing mechanism enables robust, linear pressure detection under dynamic contact conditions, while the dual‐resonant actuator delivers stable vibrotactile feedback across both low‐ and high‐frequency regimes, maintaining performance under naturally occurring contact loads. By unifying sensing and actuation through electrostatic mechanisms, the proposed interface resolves complex tactile signals in real time and enables dynamic modulation of feedback amplitude in response to pressure input, closely aligning with human tactile perception. This scalable and power‐efficient architecture provides a versatile foundation for next‐generation interactive systems, with broad implications for robotic teleoperation, immersive extended reality, and intelligent human–machine interfaces.

## 4 Experimental Section

### 4.1 Fabrication and Structural Design of the UHDRA

The unified haptic dual resonant actuator (UHDRA) was fabricated using a multilayer assembly centered on a single stainless steel (SUS 304) spring plate, in which two mechanically distinct resonant springs were monolithically patterned. The dual spring geometry was defined within the same plate by wire cutting, allowing the two flexural elements to share a common outer frame while exhibiting different effective lengths and stiffness values. This configuration enables dual resonant behavior within a single continuous structure. Detailed structural schematics and geometric parameters are provided in Figure S1. To complete the actuator stack, a printed circuit board (PCB) electrode layer coated with a polyimide (PI) film was integrated beneath the spring plate. The PI layer serves both as electrical insulation and as a compliant dielectric spacer for capacitive sensing. A metallic spacer layer was inserted to define the nominal electrode gap and to maintain mechanical stability. All layers were bonded using thin adhesive films. The total thickness of the assembled structure, including adhesive and insulation layers, was maintained below 1 mm to enable integration into thin, surface‐based touch interfaces.

### 4.2 Experimental Measurement and Analysis of Frequency‐Acceleration Response

The frequency dependent acceleration response of the UHDRA was experimentally characterized to evaluate its vibrotactile performance and to enable direct comparison with other actuator technologies. A sinusoidal voltage sweep from 20 Hz to 500 Hz was generated using a function generator (Agilent 33120A) and amplified by a high voltage amplifier (Trek 609E‐6) to drive each actuator under test. Output acceleration was measured using a piezoelectric accelerometer (PCB Piezotronics 352C33, sensitivity 97.7 mV g^−1^) mounted on the vibrating surface of each actuator. For the UHDRA, the accelerometer was attached to the top surface of the spring plate. Acceleration signals were recorded using a mixed signal oscilloscope (Tektronix MSO 2012) at a sampling rate of 2 kHz. A continuous frequency sweep covering 20–500 Hz was applied over a duration of 19 s, allowing the system to reach a quasi‐steady state response at each frequency during the sweep. The recorded time domain signals were post processed in MATLAB to extract peak to peak acceleration as a function of frequency. The same measurement protocol and data processing pipeline were applied to the comparison actuators, including a linear resonant actuator, an electromagnetic actuator, and a piezoelectric actuator.

### 4.3 Finite Element Analysis of the Dual‐Resonant Spring Structure

Finite element modeling (FEM) was performed using COMSOL Multiphysics version 6.2 (ALTSOFT, Korea, License Number: 5094592) to analyze the mechanical resonance characteristics of the dual‐resonant spring structure. The stiffness of each spring branch was tuned by adjusting the effective length of the flexural hinge to target resonant frequencies near 50 and 150 Hz. The hinge lengths were set to 26.8 mm for the low frequency branch and 23.8 mm for the high frequency branch. Modal analysis was conducted to identify the resonant frequencies and corresponding mode shapes. A frequency sweep from 20 to 300 Hz was applied under harmonic excitation conditions. The simulation captured the out of plane displacement and acceleration responses of the spring structure, providing insight into mode localization and vibration distribution across the active area.

### 4.4 Experimental Measurement of Temperature Variation of the Actuators

To evaluate the heat generation during actuator operation, the temporal temperature variation of each actuator was measured using a thermal imaging camera (FLIR ONE PRO). The experiments were conducted under laboratory conditions at 28°C, with each actuator driven at its respective resonant frequency. Prior to measurement, all actuators were conditioned for 2 h to equalize their initial temperatures, ensuring consistent experimental conditions across all samples.

### 4.5 Experimental Measurement of Capacitive Sensing Characteristics

The capacitance characteristics of the UHDRA were evaluated using a precision impedance analyzer (Agilent 4294A). Capacitance variations were recorded while applying normal forces ranging from 0 to 4 N. The measurements were performed at driving frequencies between 50 and 450 Hz with 100 Hz intervals, which were simultaneously applied to the actuator during testing. A calibrated 6‐axis force/torque sensor (AFT50) integrated into a mechanical loading stage was used to apply vertical pressure to the center of the actuator, while the capacitance data were continuously acquired through the impedance analyzer.

## Author Contributions

**Jihun Son**: conceptualization, methodology, investigation, visualization, writing – original draft, writing– review, and editing.**: Joon Hyeok Kang**: conceptualization, methodology, investigation, visualization, writing– original draft, writing– review, and editing. **Jaeha Park**: conceptualization, methodology, investigation, visualization, writing– original draft, writing – review, and editing. **Yoon‐Gi Ku**, **Yongjun Lee**, **Jun Hyeok Lee** and **Junsik Shin**.: Visualization, investigation. **Jinhyung Kim, Minjin Kim**, **Minwoo Song,** and **Gui Won Hwang**.: visualization and investigation**. Dongbum Pyo, Gwanghyun Jo,** and **Jeong‐Hoi Koo**: investigation. **Young‐Min Kim**: supervision, conceptualization, writing‐ review, and editing. **Changhyun Pang**: supervision, conceptualization, methodology, writing‐ original draft, writing‐ review, and editing. **Tae‐Heon Yang**: supervision, conceptualization, methodology, writing‐ original draft, writing‐ review, and editing.

## Conflicts of Interest

The authors declare no conflicts of interest.

## Supporting information




**Supporting File 1**: advs75103‐sup‐0001‐SuppMat.docx.

**Supporting File 2**: advs75103‐sup‐0002‐MovieS1.mp4.

**Supporting File 3**: advs75103‐sup‐0003‐MovieS2.mp4.

**Supporting File 4**: advs75103‐sup‐0004‐MovieS3.mp4.

**Supporting File 5**: advs75103‐sup‐0005‐MovieS4.mp4.

## Data Availability

The data that support the findings of this study are available from the corresponding author upon reasonable request.
