# Supplementary material for: A Large‐Area Broadband Multimodal Dual‐Resonant Haptic Device for Bidirectional Telerobotic and Augmented Interactions
Source: Adv Sci (Weinh). 2026 Apr 7;13(34):e75103. doi: 10.1002/advs.75103 (PMC13285133; doi:10.1002/advs.75103)
Supplement: Supplementary file 1 — Supporting File 1: advs75103‐sup‐0001‐SuppMat.docx. [file ADVS-13-e75103-s002.docx]

Supporting Information

**A Large-Area Broadband Multimodal Dual-Resonant Haptic Device for Bidirectional Telerobotic and Augmented Interactions**

*Jihun Son^†^, Joon Hyeok Kang^†^, Jaeha Park^†^, Yoon-Gi Ku, Yongjun Lee, Jinhyung Kim, Minjin Kim, Jun Hyeok Lee, Junsik Shin, Minwoo Song, Gui Won Hwang, Dongbum Pyo, Gwanghyun Jo, Jeong-Hoi Koo, Young-Min Kim*, Changhyun Pang*, Tae-Heon Yang**

J. Son, J. Kim, M. Kim, M. Song, G. W. Hwang, and Prof. C. Pang

School of Chemical Engineering, Sungkyunkwan University (SKKU), 2066 Seobu-ro, Jangan-gu, Suwon, Gyeonggi-do 16419, Republic of Korea

J. H. Kang, J. Park, Y.-G. Ku, Y. Lee, J.-H. Lee, J. Shin, and Prof. T.-H. Yang

Department of Mechanical Engineering, Konkuk University, Seoul 05029, Republic of Korea

Dr. D. Pyo

Human-Centric Robotics R&D Department, Korea Institute of Industrial Technology, Ansan-si, Gyeonggi-do, 15588, Republic of Korea

Prof. G. Jo

Department of Mathematical Data Science, Hanyang University ERICA, 55Hanyangdaehak-ro, Ansan-si, Gyeonggi-do 15588, Republic of Korea

Prof. J.-H. Koo

Department of Mechanical and Manufacturing Engineering, Miami University, Oxford, OH, USA

Dr. Y.-M. Kim.

R&D Strategy Division, Korea Institute of Oriental Medicine (KIOM) 1672 Yuseongdaero, Yuseong-gu, Daejeon, 34054 Republic of Korea

Prof. C. Pang

Samsung Advanced Institute for Health Sciences and Technology (SAIHST), Sungkyunkwan University, Suwon, Gyunggi-do 16419, Republic of Korea

[^†^] J. S., J. H. K., and J. P. contributed equally to this work.

*Corresponding author. E-mail: irobo77@kiom.re.kr (Y.-M. K) ORCID: 0000-0001-7417-5290, chpang@skku.edu (C.P.) ORCID: 0000-0001-8339-7880, and thyang@konkuk.ac.kr (T.-H. Y.) ORCID: 0000-0003-4316-4323

**Note** **S1. Calculation of center delivery**

Spatial localization was quantified from the measured one-dimensional acceleration profile along the scan coordinate. The scan range was divided into three regions: a central band centered at the geometric midpoint of the scan, and two edge bands at the left and right ends. The central and edge bands used the same band width, defined either as a fixed fraction of the total scan length or as a fixed physical length, and this choice was kept identical across all conditions. For each mode, we first took the magnitude of acceleration to avoid sign cancellation. We then computed the band averaged acceleration in the central band and in the edge region. The edge value was obtained by averaging the left and right edge bands and combining them, rather than treating the edges as one continuous interval. Finally, center delivery (%) was defined as the central band average divided by the sum of the central and edge averages, multiplied by 100. A higher center delivery indicates that vibration is more concentrated near the center, while a lower value indicates a more edge weighted spatial distribution.

**Note S2: Analysis method for measuring resonance point, and vibration acceleration**

When touch or pressure is applied to the haptic interface integrated with the UHDRA, the distance between the internal electrodes and the spring structure changes, resulting in a variation in capacitance. This capacitance change is converted into digital data by a capacitance-to-digital converter (CDC) and transmitted to a microcontroller unit (MCU). Based on the sensed capacitance information, the MCU generates control signals for haptic feedback. In this process, GPIO1 controls the operation of the high-voltage (HV) power supply through an isolated gate driver, while GPIO2 directly drives the MOSFETs in the HV switching stage to regulate the discharge timing at the output node. Through this high-voltage switching operation, a time-modulated high-voltage waveform is generated at the output node and applied to the actuator electrodes. As a result, the user perceives vibration-based haptic feedback that corresponds to the applied touch and pressure.

**Note S3. Frequency reproduction analysis of the UHDRA and LRA using aperiodic waveforms**

Two aperiodic vibration signals with dominant frequencies of approximately 50 Hz and 150 Hz were employed to evaluate the frequency reproduction accuracy of the UHDRA and the LRA. The reference signals were obtained by scratching sandpapers of varying surface roughness levels using a needle equipped with an accelerometer. Their dominant frequencies were confirmed through fast Fourier transform (FFT) analysis in MATLAB. To minimize transient effects, the stable portion of each 4 s signal (1-3 s) was selected for analysis. The selected data were divided into overlapping Hamming windows, and spectrograms were computed to identify the dominant frequency components. The frequency corresponding to the maximum spectral amplitude within each window was extracted and averaged to obtain the representative frequency of the signal. The two selected aperiodic vibration signals were then used as input waveforms for both the UHDRA and the LRA, and the resulting vibration responses were recorded using an oscilloscope. The actuator responses were analyzed using the same procedure, and the results were compared to evaluate the fidelity of frequency reproduction.

**Note S4: Measurement of the rising time and falling time of the actuators**

A time-limited sinusoidal input signal was applied to each actuator to observe both transient and steady-state vibration responses. This signal configuration allowed clear identification of the rising phase, corresponding to the amplitude buildup, and the falling phase, representing the decay after excitation. The transient response characteristics of four actuators (LRA, Electromagnetic actuator, Piezoelectric actuator, and UHDRA) were experimentally evaluated to compare their rising and falling times. Each actuator was driven at its respective resonant frequency using the same time-limited sinusoidal input. The driving signal was generated by a function generator (Agilent 33120A) and amplified by a high-voltage amplifier (Trek 609E-6), while the output acceleration was measured using a high-sensitivity accelerometer (PCB Piezotronics 352C33) and recorded with a digital oscilloscope (Tektronix MSO 2012). The rising time (tᵣ) was defined as the duration required for the vibration amplitude to increase from 10% to 90% of the steady-state value after excitation, whereas the falling time (tf) was defined as the interval for the amplitude to decay from 90% to 10% of that value after the signal termination. Both parameters were extracted from the acceleration envelope using Hilbert transform analysis in MATLAB.

**Note S5. Determination of the damping ratio from free-decay vibration response**

The damping ratio of the actuator was evaluated from its free-decay vibration response using the logarithmic decrement method. After termination of the excitation signal, the actuator exhibits an underdamped oscillatory response with an exponentially decaying amplitude.

The logarithmic decrement, δ, was calculated from the ratio of successive peak amplitudes in the time-domain acceleration signal:

$$\delta=\frac{1}{n}\ln\left( \frac{y_{1}}{y_{n+1}} \right),$$

where $y_{1}$and $y_{n+1}$denote the amplitudes of two peaks separated by $n$oscillation cycles. To reduce the influence of measurement noise, multiple cycles ($n\geq3$) were used for the calculation.

The damping ratio, ζ, was then obtained from the logarithmic decrement as

$$\zeta=\frac{\delta}{\sqrt{\left( 2\pi)^{2} + \delta^{2} \right.}}.$$

For lightly damped systems ($\zeta\ll1$), which is applicable in this study, the damping ratio can be approximated by

$$\zeta\approx\frac{1}{2\pi n}\ln\left( \frac{y_{1}}{y_{n+1}} \right).$$

This time-domain approach enables reliable estimation of damping characteristics without requiring frequency-domain measurements and is widely adopted for experimental characterization of underdamped mechanical systems. All damping ratios reported in this work were extracted from the free-decay region of the vibration response to ensure consistency and accuracy.

**Note S6: Measurement of the power consumption of the actuators**

The electrical power consumption P of each actuator was evaluated from the measured root-mean-square (RMS) voltage $V_{rms}$, current $I_{rms}$, and the phase angle θ between them as:

$$P=V_{rms}\cdot I_{rms}\cdot cos\theta$$

This general expression was applied to the linear resonant actuator (LRA) and electromagnetic actuator, which behave predominantly as resistive-inductive loads. For capacitance-based actuators, including the UHDRA and the piezoelectric actuator, dielectric loss was considered, and the real power was alternatively expressed as:

$$P=2\pi\cdot f\cdot C\cdot tan\delta\cdot{V_{rms}}^{2}$$

where f is the driving frequency, C is the capacitance, and tanδ is the dielectric loss tangent. All measurements were performed at the resonant frequency of each actuator to ensure accurate comparison of power consumption under their maximum operating efficiency conditions.

**Note** **S7. Signal process of bidirectional system.**

When a user applies touch or pressure to the haptic interface integrated with the UHDRA, the distance between the internal electrode and the spring within the UHDRA changes, resulting in a variation in capacitance. This capacitance change is converted into digital data by a capacitance-to-digital converter (CDC) and transmitted to a microcontroller unit (MCU). Based on the sensed capacitance information, the MCU generates control signals for haptic feedback. Specifically, GPIO1 regulates the operation of the high-voltage (HV) power source via MOSFETs, while GPIO2 controls the MOSFET constituting the HV switching stage via an isolated gate driver to precisely manage the discharge timing at the output node and thereby realize high-voltage switching. Through this high-voltage switching process, a temporally modulated high-voltage waveform is generated at the output node and applied to the actuator electrodes, enabling the user to perceive vibrotactile feedback corresponding to the applied touch and pressure.

**Note S8: Method for measuring resonance frequency shift of the UHDRA under varying contact forces**

To investigate the resonance frequency shift of the UHDRA under different contact forces, a 3-axis force-controlled indentation system consisting of a three-axis stage, an indenter, and a 6-axis force/torque sensor was employed. Normal forces ranging from 0 N to 2 N were linearly applied to the actuator, and its resonant characteristics were subsequently analyzed. As the applied force increased, the resonance frequency of the low-resonance actuator shifted from approximately 50 Hz to 40 Hz, while that of the high-resonance actuator decreased from 150 Hz to 140 Hz.

**Note S9: Experimental measurement of vibration propagation characteristics**

To evaluate the uniformity of vibration propagation, the vibration distribution of the UHDRA and a conventional linear resonant actuator (LRA) was measured across 36 sensing regions arranged in a $6 \times6$ grid over the touchpad surface. Each region corresponded to an independent measurement point with an area of approximately 8 mm × 8 mm, ensuring consistent spatial resolution across the surface. To maintain comparable experimental conditions, two LRAs were mounted beneath the touchpad surface in a vertical orientation to replicate the dual-spring configuration of the UHDRA. For both actuators, the mean vibration amplitude across the 36 grid points was calculated, and the deviation of each point from this mean value was mapped to visualize the spatial uniformity of vibration propagation over the touchpad. This analysis provided a direct comparison of how evenly vibration energy was distributed across the surface in each actuator configuration.

**Note S10: Evaluation of spatial uniformity across the 36-grid array.**

To provide a more in-depth evaluation of the uniformity metric, the Coefficient of Variation (CV) was employed to quantitatively compare the dispersion of the LRA array and the UHDRA. The Coefficient of Variation is a statistical metric commonly used to compare the relative variability among different datasets, and in this study it was used to quantitatively evaluate the 2D uniformity of the LRA array and UHDRA. In addition, to analyze uniformity along specific directions, further analyses were conducted along six horizontal paths (e.g., P1 → P6, P7 → P12) and two diagonal paths. The coefficient of variation is defined as follows:

$$CV=\frac{\sigma}{\mu} \times100(\%)$$

Here, $\mu$represents the mean of the population and $\sigma$represents the standard deviation. The mean $\mu$and standard deviation $\sigma$of the population can be expressed as:

$$\mu=\frac{\sum_{i=1}^{n} x_{i}}{n}$$

where $x_{i}$denotes the vibration intensity measured at the $i$-th grid and $n$is the total number of grids in the population.

$$\sigma=\sqrt{\frac{\sum_{i=1}^{n} {(x_{i}-\mu)}^{2}}{n}}$$

Using this approach, the Coefficient of Variation for the 36 sensing regions was compared between the LRA array and the UHDRA, and the mean and standard deviation along eight defined paths were further analyzed to evaluate the quantitative difference in dispersion between the two systems. The analysis of spatial variability at individual positions shows that the dispersion of the LRA array is approximately three times larger than that of the UHDRA. Furthermore, in the full-area uniformity analysis defined by the eight paths, the LRA exhibits a wide range of mean values (μ ≈ 40-80), whereas the UHDRA shows a much narrower distribution (μ ≈ 80-90), indicating consistently high uniformity across all paths.

**Note S11: Measurement setup and data acquisition for bidirectional haptic teleoperation**

The position and force applied by the user on the touchpad were acquired by a microcontroller unit (MCU). At the initial stage, the robotic arm was activated by a click input, which triggered its movement to the commanded starting position. Subsequently, the arm was continuously controlled on a two-dimensional plane according to the user’s touch and pressure inputs detected by the UHDRA. These signals were converted into digital commands and transmitted to the robotic arm to generate corresponding movements. During operation, variations in motor current were measured and sent back to the MCU as feedback data. Based on this feedback, pre-programmed vibration waveforms were generated and delivered to the user through the UHDRA to provide tactile feedback. For the robotic platform, a PiPER 6-DOF robotic arm (AgileX Robotics, China) was employed.

**Note S12: Experimental implementation of haptic feedback for diverse user commands**

The haptic feedback performance of the UHDRA was experimentally evaluated under eleven representative user interaction scenarios, simulating a typical laptop usage environment. Haptic signals corresponding to events such as Button Click and Double Click were generated by directly measuring the acceleration responses of an actual physical touchpad (MSI Prestige 15 A11UC) equipped with an accelerometer during finger pressing actions. For dynamic gestures such as Zoom-In and Zoom-Out, vibration patterns were designed to gradually increase and decrease in intensity, respectively, capturing the intuitive tactile sensation associated with these interactions. Following this approach, a total of eleven distinct haptic feedback signals were constructed to represent various user interaction conditions. These reference signals were then applied as input to the UHDRA, and the resulting output accelerations were recorded using an accelerometer to evaluate the actuator’s reproduction fidelity.

**Note S13: Method for evaluating vibration output roughness of the UHDRA**

To quantitatively evaluate the surface roughness characteristics of vibration outputs generated by the UHDRA, an analytical method was developed based on the correlation between waveform morphology and perceived texture. Two primary features were extracted from the measured vibration signals: waveform roughness $(X_{1})$ and dominant frequency $(X_{2})$. These parameters were identified as key indicators of the perceived surface roughness parameter $R_{a}$.

The relationship between vibration features and surface roughness was expressed as:

$$R_{a}\left( X_{1},X_{2} \right)=AX_{1}+\frac{B}{X_{2}-C}+D$$

where *A, B, C,* and *D* represent empirical coefficients obtained by least-squares fitting of the measured data. The derived model was then applied to the vibration signals produced by the UHDRA to reconstruct the correlation surface of $R_{a}(X_{1},X_{2})$. The reconstructed results exhibited strong consistency between the measured and predicted roughness values, confirming that the proposed method effectively evaluates and characterizes the vibration output roughness of the UHDRA in terms of tactile texture perception.

**Note S14: Construction of augmented multisensory haptic signal**

To construct augmented multisensory haptic signals applicable to virtual texture rendering, acceleration data were collected from ten different types of sandpaper with distinct surface roughness values. Each vibration waveform was analyzed extracting two characteristic parameters: waveform roughness $X_{1}$and dominant frequency $X_{2}$. The reconstructed relationship $R_{a}(X_{1},X_{2})$ was then applied to identify vibration patterns corresponding to specific target textures (dolphin skin, coral, and turtle shell). The reference $R_{a}$ values for these biological surfaces were obtained from previously published studies. Among the ten sandpapers, those exhibiting the closest predicted roughness values to the reference $R_{a}$ values were selected as the representative sources for each texture. The selected acceleration signals were digitized and normalized to ensure amplitude consistency across stimuli.

**Note S15: Augmented multisensory haptic feedback process in virtual space**

A laptop-based virtual interaction system integrating the UHDRA was developed to realize augmented multisensory haptic feedback in a virtual environment. The UHDRA module was embedded beneath the touchpad surface, enabling simultaneous tactile actuation and capacitive force sensing. When a user touched or pressed images of a dolphin, coral, or turtle displayed on the laptop screen, the input was detected by the microcontroller unit (MCU) through capacitance variations in the UHDRA. The MCU identified the selected image, selected the corresponding haptic feedback signal. The generated signal was amplified and applied to the UHDRA to produce texture-specific vibration patterns corresponding to each visual element. Concurrently, real-time capacitance changes were continuously monitored and calibrated, allowing the feedback intensity to vary proportionally with the user’s applied force. This bidirectional interaction enabled users to distinctly perceive differences in tactile intensity and texture.


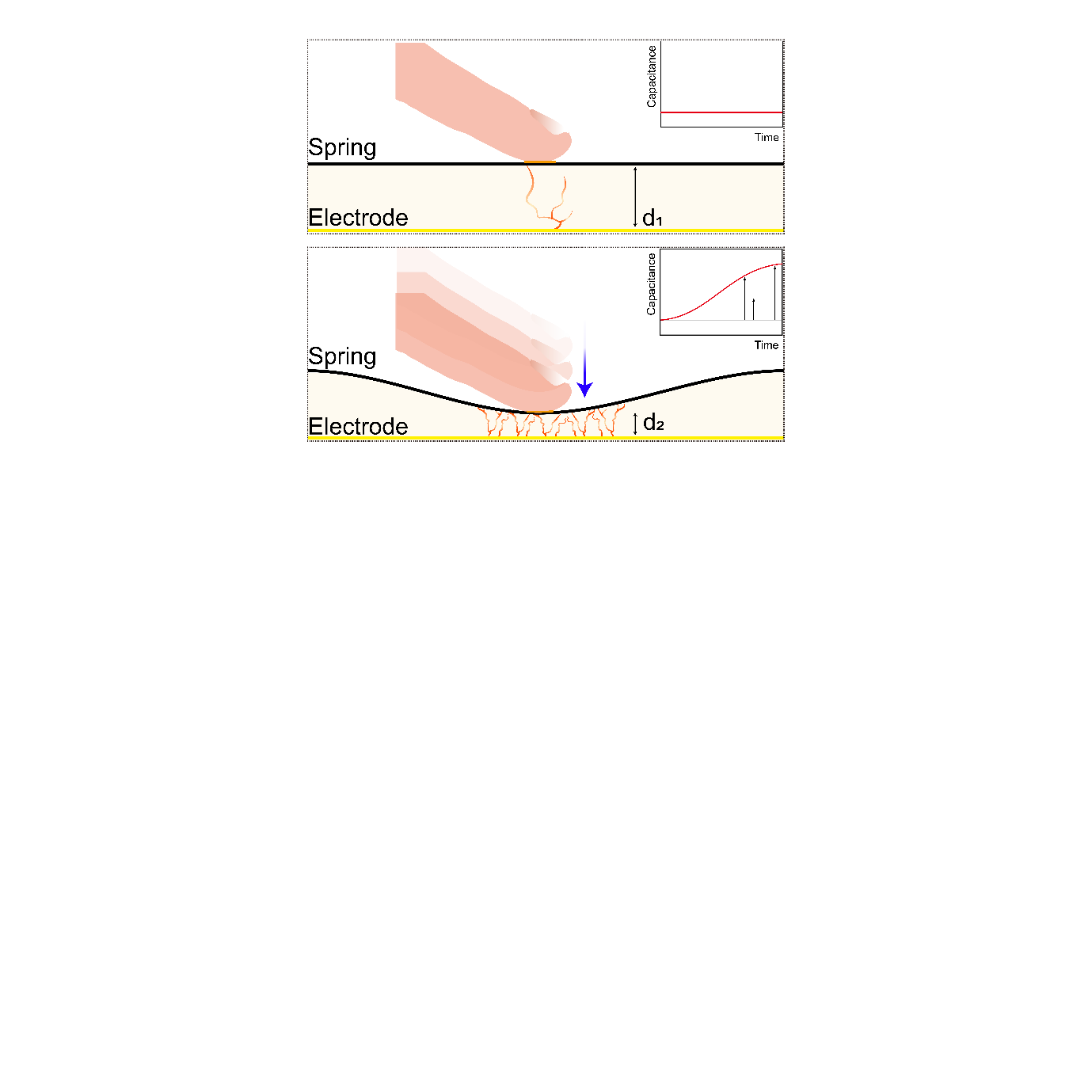


Figure S1. Schematic illustration of the capacitive sensing principle.

The upper image shows the initial state with finger contact, where the capacitance remains constant at a baseline distance d₁. The lower image shows the pressed state, where spring deformation increases the separation to d₂, producing a time-varying capacitance that enables pressure sensing.


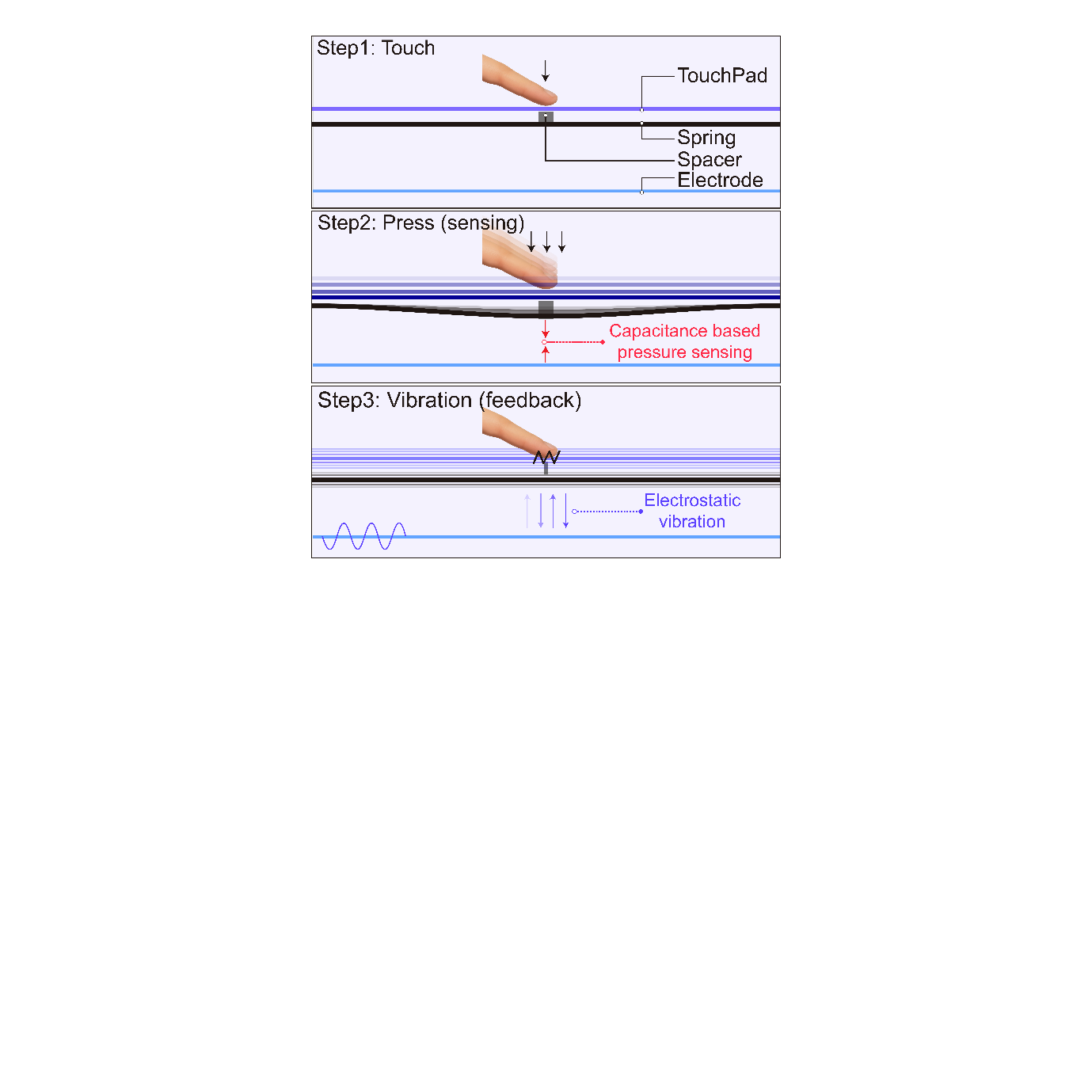


Figure S2. Operation principle of the UHDRA.

The top image shows a finger contacting the surface, followed by spring deformation that modulates electrode spacing for capacitance-based pressure sensing. The final image illustrates electrostatic actuation generating vibrotactile feedback to the fingertip.

***
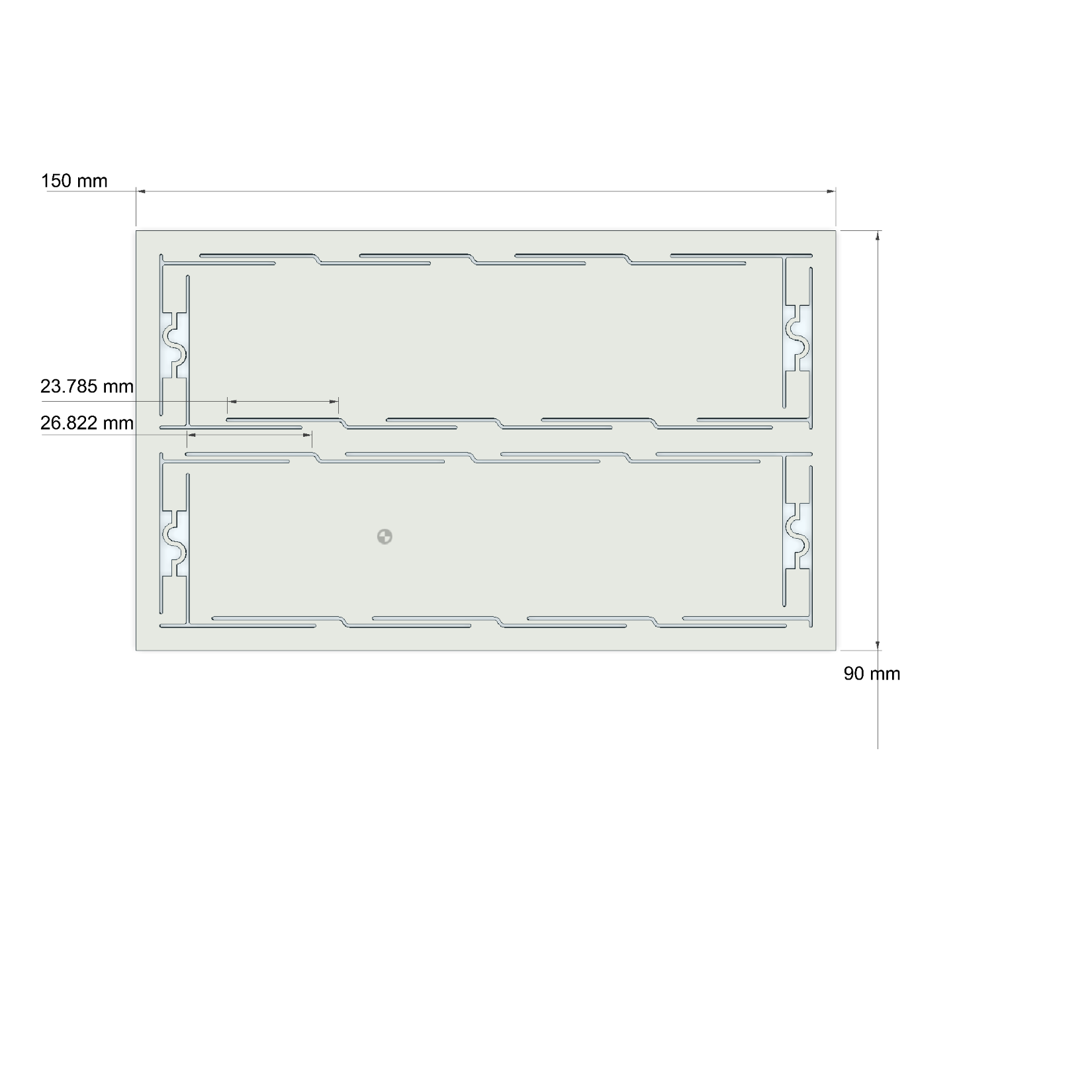
***

Figure S3. CAD model of the dual-resonant spring design.

The CAD schematic illustrates a stainless steel (SUS) plate with overall dimensions of 150 mm × 90 mm, containing two leaf-spring architectures with different effective lengths of 26.822 mm and 23.785 mm. The longer length exhibits lower stiffness and enables low-frequency resonance, while the shorter length provides higher stiffness and support high-frequency resonance.


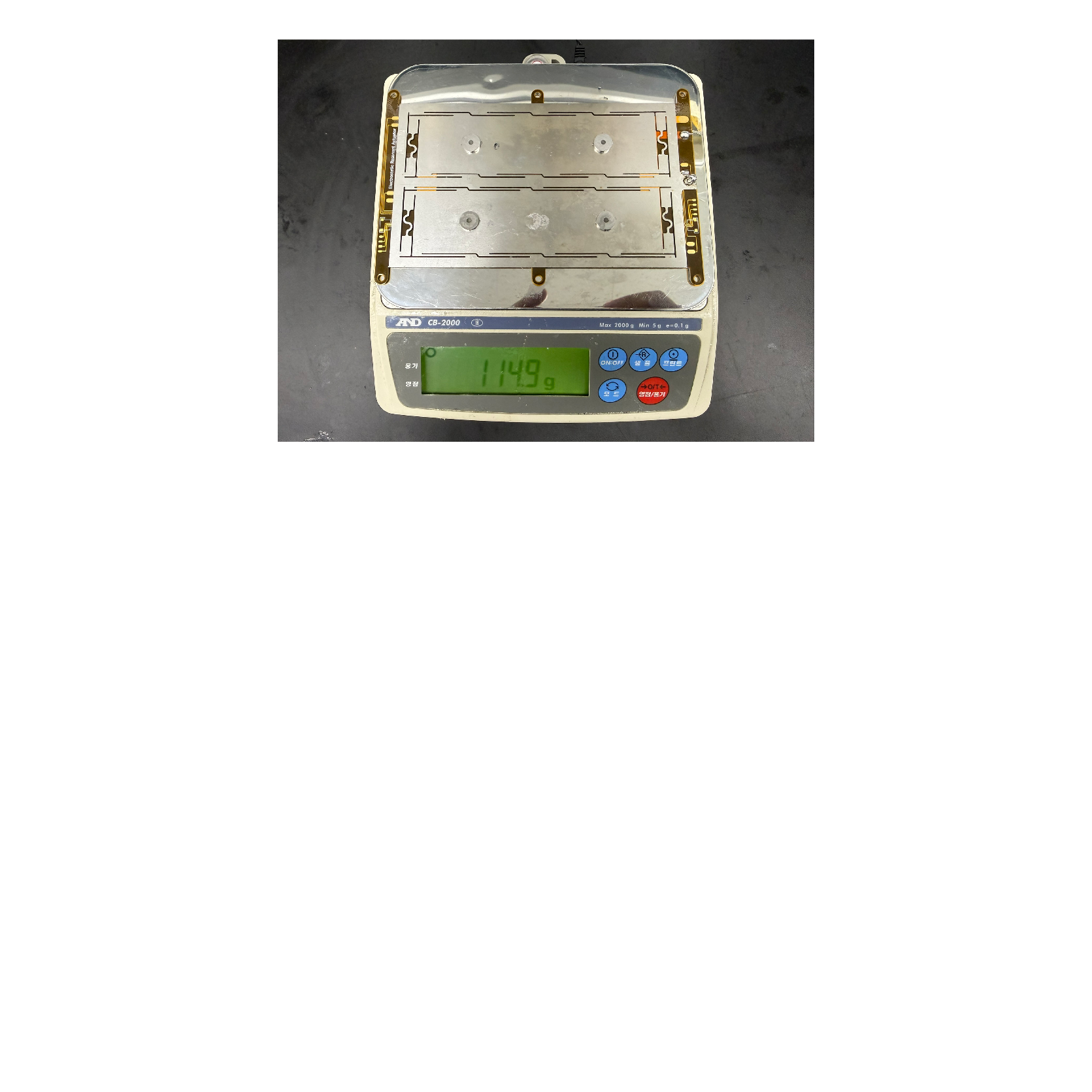


Figure S4. Weight of the UHDRA integrated with electrode.


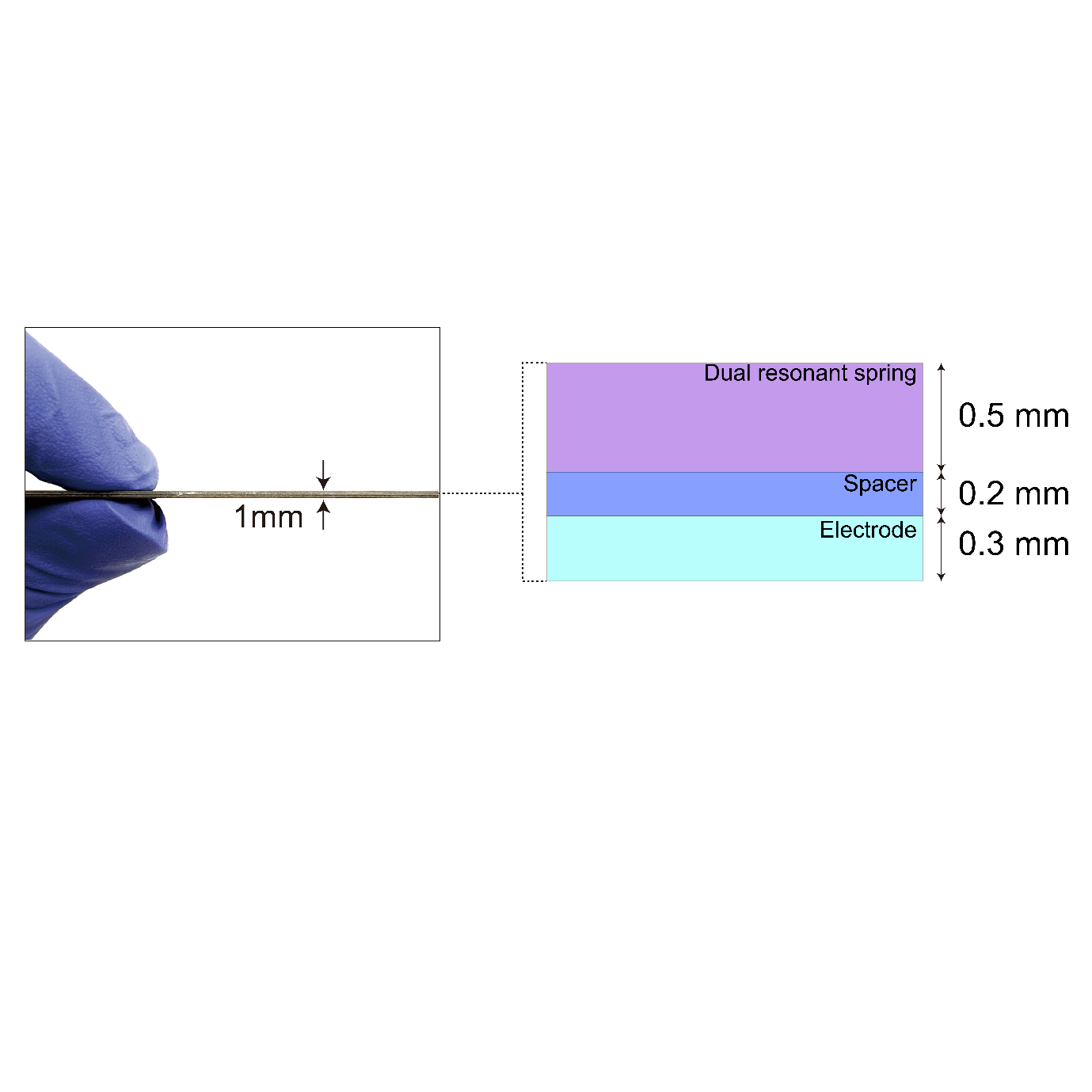
**Figure S5. The thickness of UHDRA.** (Left) Photograph showing the overall UHDRA thickness of 1 mm. (Right) Schematic of the layered architecture, consisting of a dual-resonant spring (0.5 mm), a spacer (0.2 mm), and an electrode (0.3 mm).


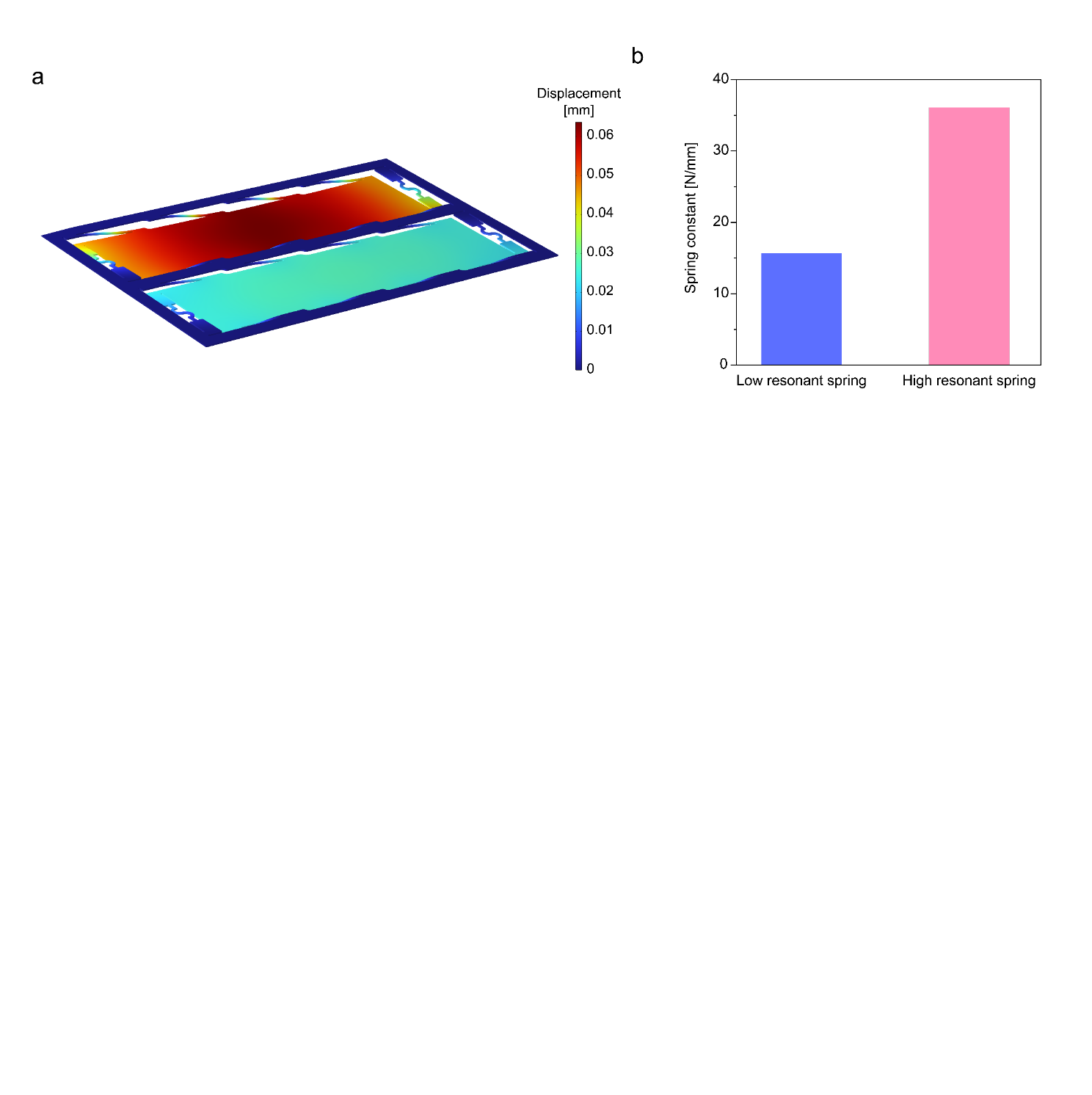


**Figure S6.** **Extraction of spring constants for the resonant spring structures.** (a) Displacement profile under applied loading, showing mode-dependent deformation of the spring geometry extracted by COMSOL simulation (b) Extracted Spring constants of the low-resonance spring (≈15 N/mm) and the high-resonance spring (≈35 N/mm).

Table S1. Comparison of vibrotactile frequency bandwidth

| Actuator type | Frequency bandwidth range | ΔFrequencey bandwidth | Ref. number |
| --- | --- | --- | --- |
| Solenoid actuator | 80 - 110 | 30 | [1] |
| Linear resonant actuator | 193 - 207 | 14 | [2] |
| VCM | 50 - 90 | 40 | [3-5] |
| Piezoelectric actuator | 232 - 236 | 4 | [6, 7] |
| UHDRA | 30 - 70 / 130 - 170 | 80 | Our work |

For each device, vibration response is measured under frequency sweep excitation, and the usable bandwidth is defined as the frequency range within the -3 dB points relative to the peak acceleration at resonance.


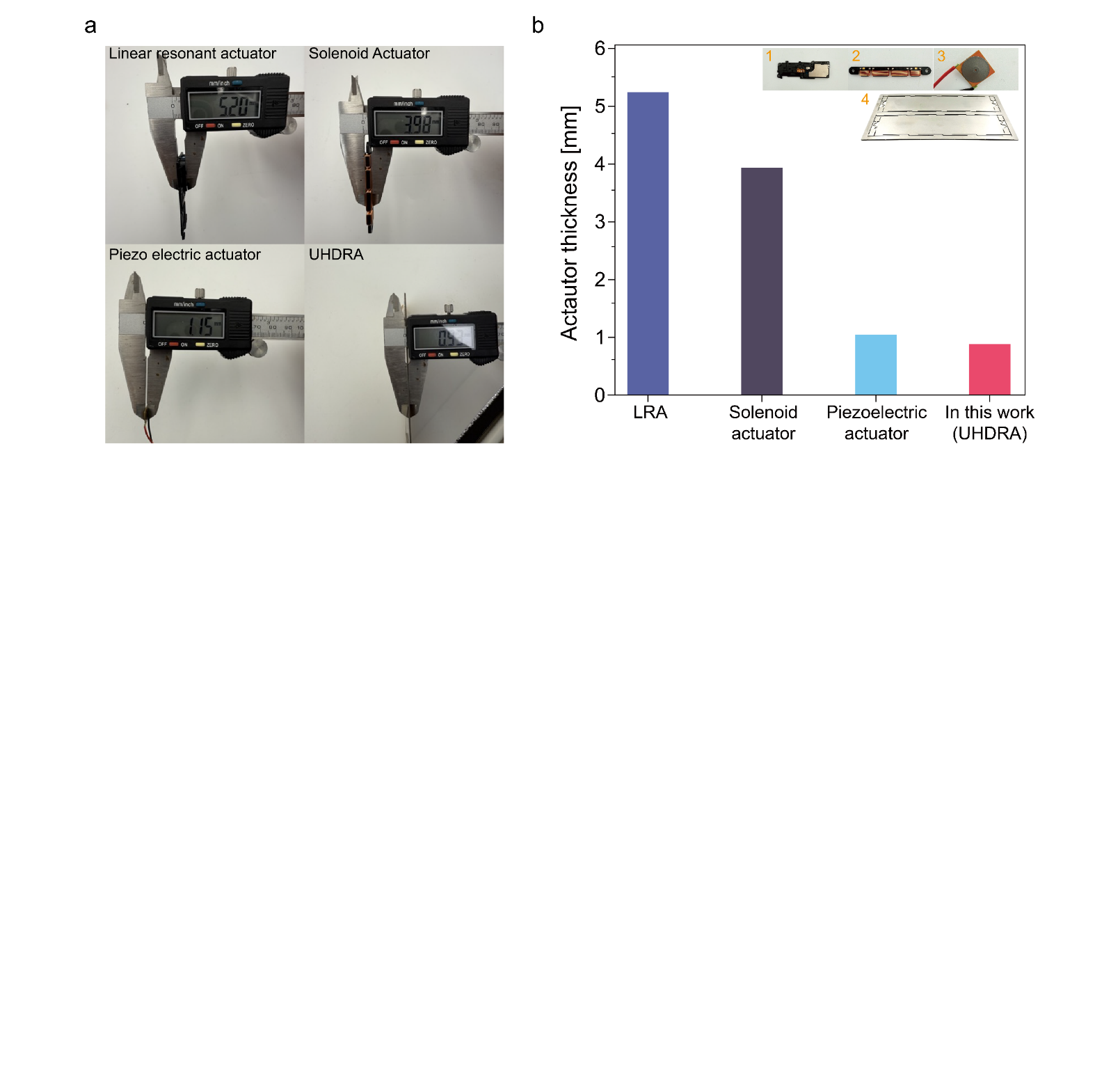


Figure S7. Comparison of actuator thickness with other actuator types. (a) Photographs of thickness for three commercial actuators and the UHDRA using a vernier caliper. The linear resonant actuator (LRA) was obtained from a Galaxy S20 Ultra smartphone vibration module. The solenoid-type actuator was obtained from a MacBook trackpad haptic module. The piezoelectric actuator was a TDK InvenSense PowerHap™ piezoelectric haptic actuator (B54102H1020A001). (b) Measured thickness of the actuator.

| Actuator type | Acceleration [g] | Power [W] | Area [mm^2^] | Thickness [mm] | Ref. number |
| --- | --- | --- | --- | --- | --- |
| LRA | 4.3 | 0.32 | 348.5 | 6.2 | [2] |
|  | 1.5 | 0.3625 | 78.54 | 4 | [8] |
|  | 3 | 0.3 | 99 | 3.2 | [9] |
|  | 1.2 | 0.2 | 78.54 | 3.4 | [10] |
|  | 0.905 | 0.135 | 50.27 | 3.3 | [11] |
|  | 1.5 | 0.13 | 78.54 | 3.7 | [12] |
|  | 1 | 0.14 | 50.27 | 3.25 | [13] |
|  | 1.5 | 0.18 | 154 | 2.5 | [13] |
| ERM | 1.4 | 0.195 | 70.87 | 3 | [14] |
|  | 0.6 | 0.246 | 38.48 | 2 | [11] |
| Solenoid actuator | 46 | 2.7 | 270.1 | 12.75 | [15] |
| Piezoelectric actuator | 5.2 | 0.19 | 90.25 | 1.25 | [11] |
| VCM | 8 | 1.18 | 137 | 37.69 | [16] |
| UHDRA | 2 | 0.038 | 11200 | 0.99 | Our work |

Table S2. Comparison of acceleration efficiency as a function of area-to-thickness ratio

**
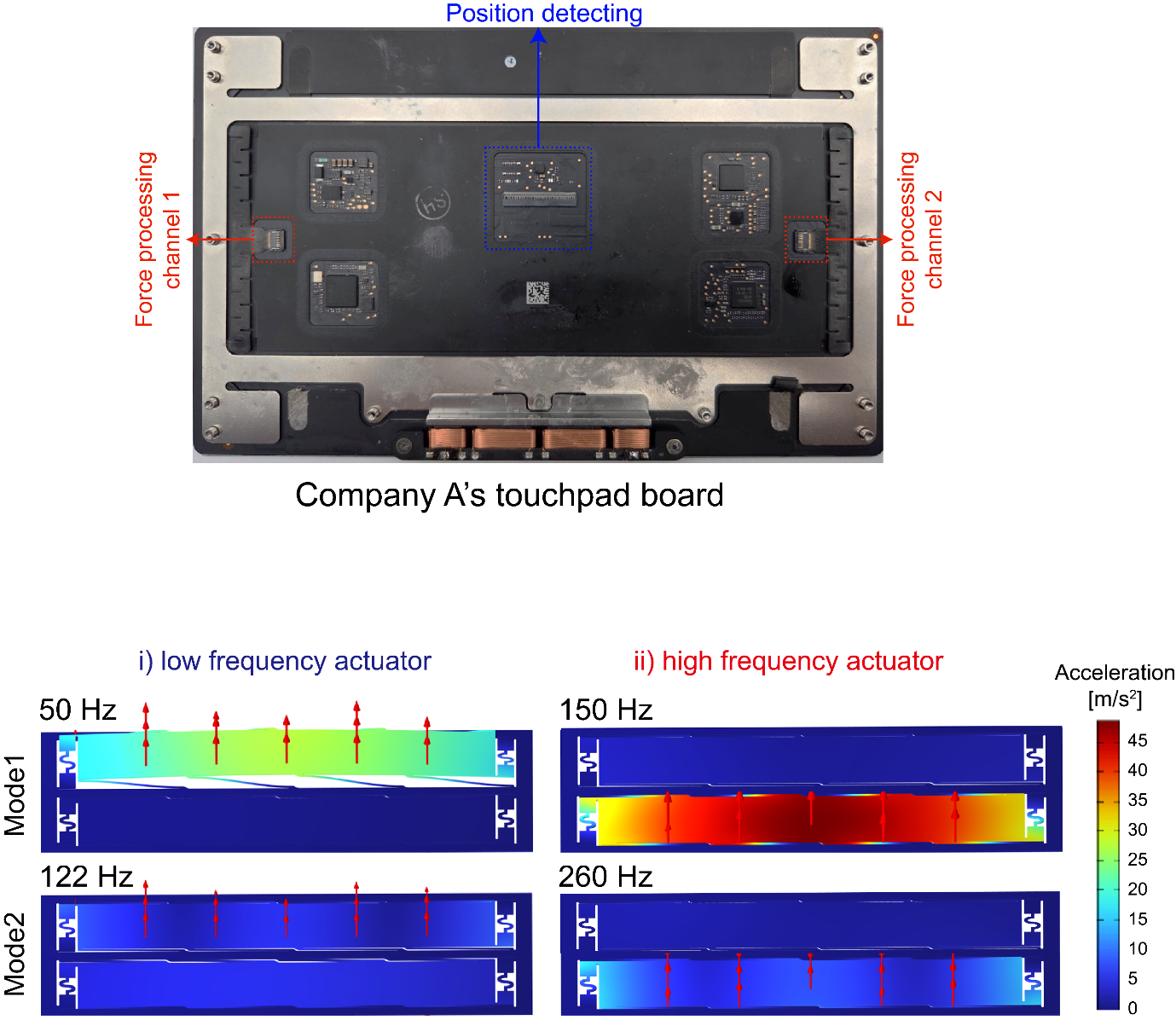
 Figure S8. Eigenmode analysis of dual-resonant actuators by COMSOL simulation showing the dominant vibration modes and motion directionality.** The low-frequency actuator exhibits resonance modes at 50 Hz and 122 Hz, while the high-frequency actuator shows modes at 150 Hz and 260 Hz. The red arrows indicate the dominant out-of-plane motion of the structure.


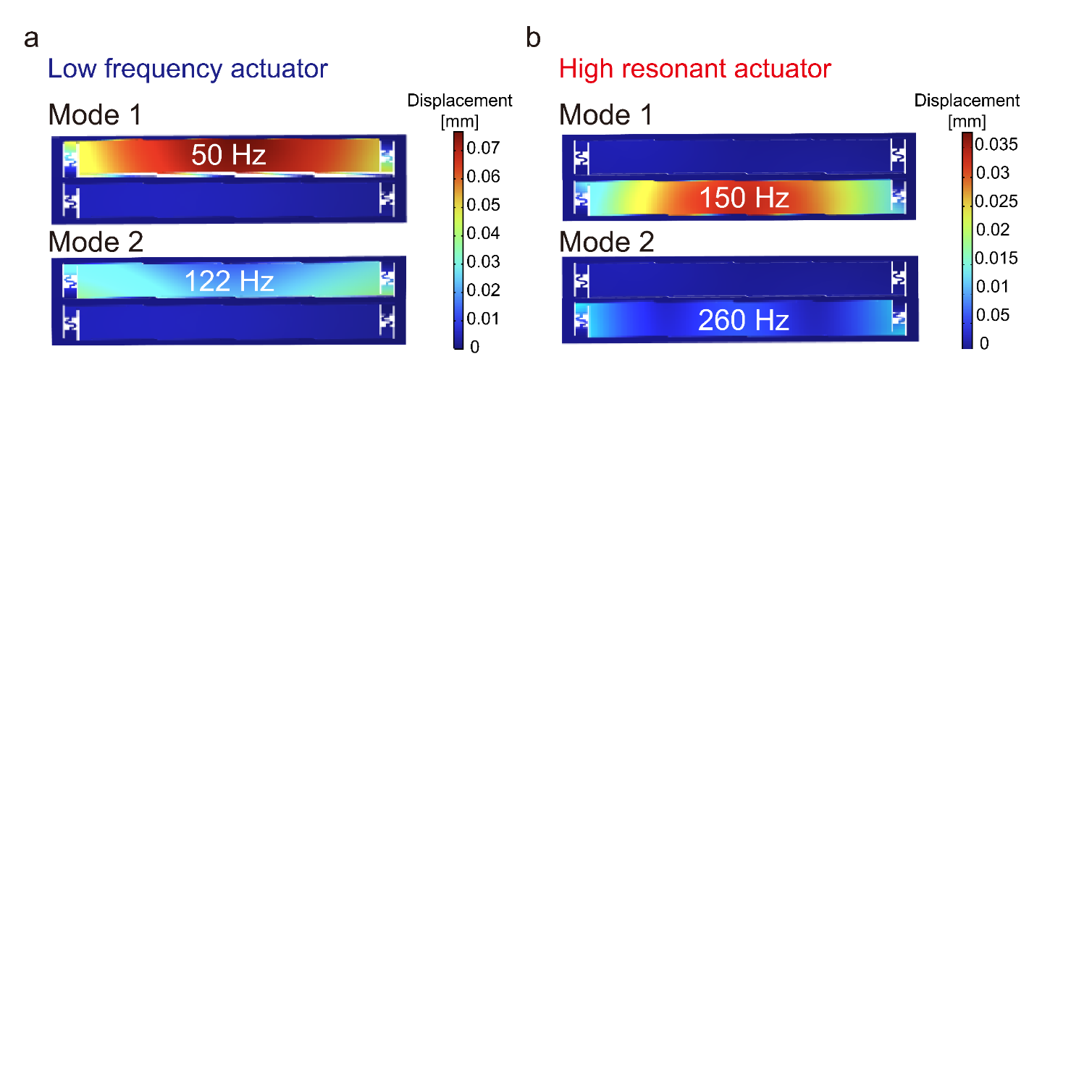
Figure S9. Eigenmode analysis of dual-resonant actuators by COMSOL simulation. (a) Mode shapes of the low-frequency actuator. Mode 1 occurs at 50 Hz, characterized by large out-of-plane displacements concentrated near the central region, whereas Mode 2 appears at 122 Hz, showing distributed nodal regions across the structure. Color mapping indicates relative displacement magnitude. (b) Mode shapes of the high-frequency actuator. Mode 1 is observed at 150 Hz, and Mode 2 at 260 Hz, both exhibiting smaller displacements compared to the low-frequency actuator due to higher stiffness associated with shorter spring geometry. The contrast between (a) and (b) highlights the effect of spring length on resonance behavior and validates the dual-resonant design for multimodal haptic output.


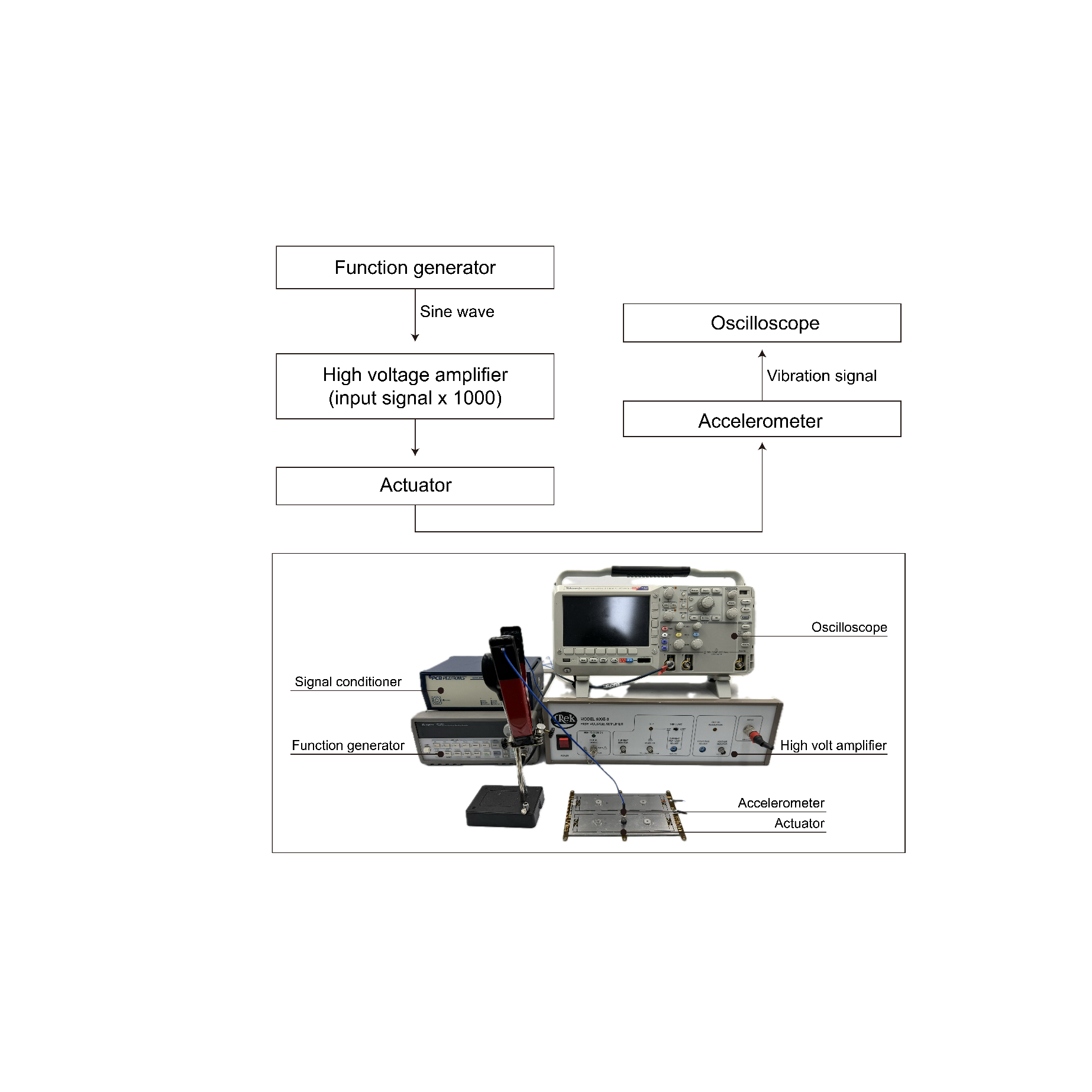


**Figure S10. Experimental setup for acceleration measurement.** An accelerometer was attached to the surface of the actuator to detect vibration. A sinusoidal input signal was generated from a function generator, amplified through a high-voltage amplifier, and applied to the actuator. The resulting vibration was captured by the accelerometer and transferred to the oscilloscope for recording and analysis. The same setup was used to measure the UHDRA and three commercial reference actuators, including an LRA from a Galaxy S20 Ultra vibration module, a solenoid-type actuator from a MacBook trackpad haptic module, and a piezoelectric actuator (TDK InvenSense PowerHap, B54102H1020A001). This setup allowed reliable acquisition of acceleration signals as a function of driving frequency, ensuring accurate evaluation of the actuator’s resonant performance.


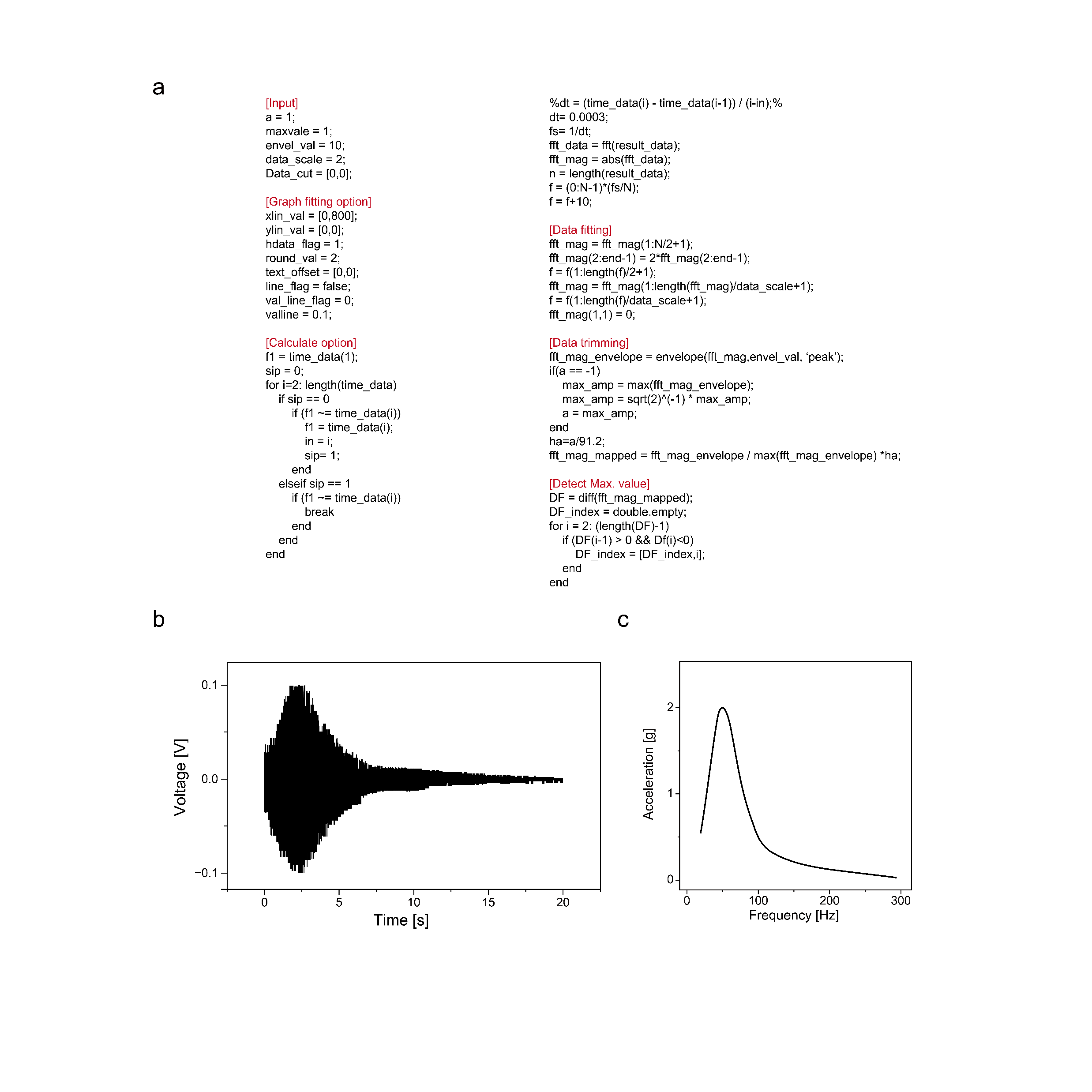


Figure S11. Conversion of vibration sweep data (10-300 Hz) into frequency-acceleration domain using MATLAB. (a) MATLAB code implementing FFT and data filtering. (b) Measured vibration sweeps signal ranging from 10 Hz to 300 Hz. (c) Converted frequency-acceleration spectrum obtained using the MATLAB code, demonstrating the ability to extract frequency-dependent acceleration amplitudes, which are later used to evaluate the resonant characteristics of the UHDRA actuator.


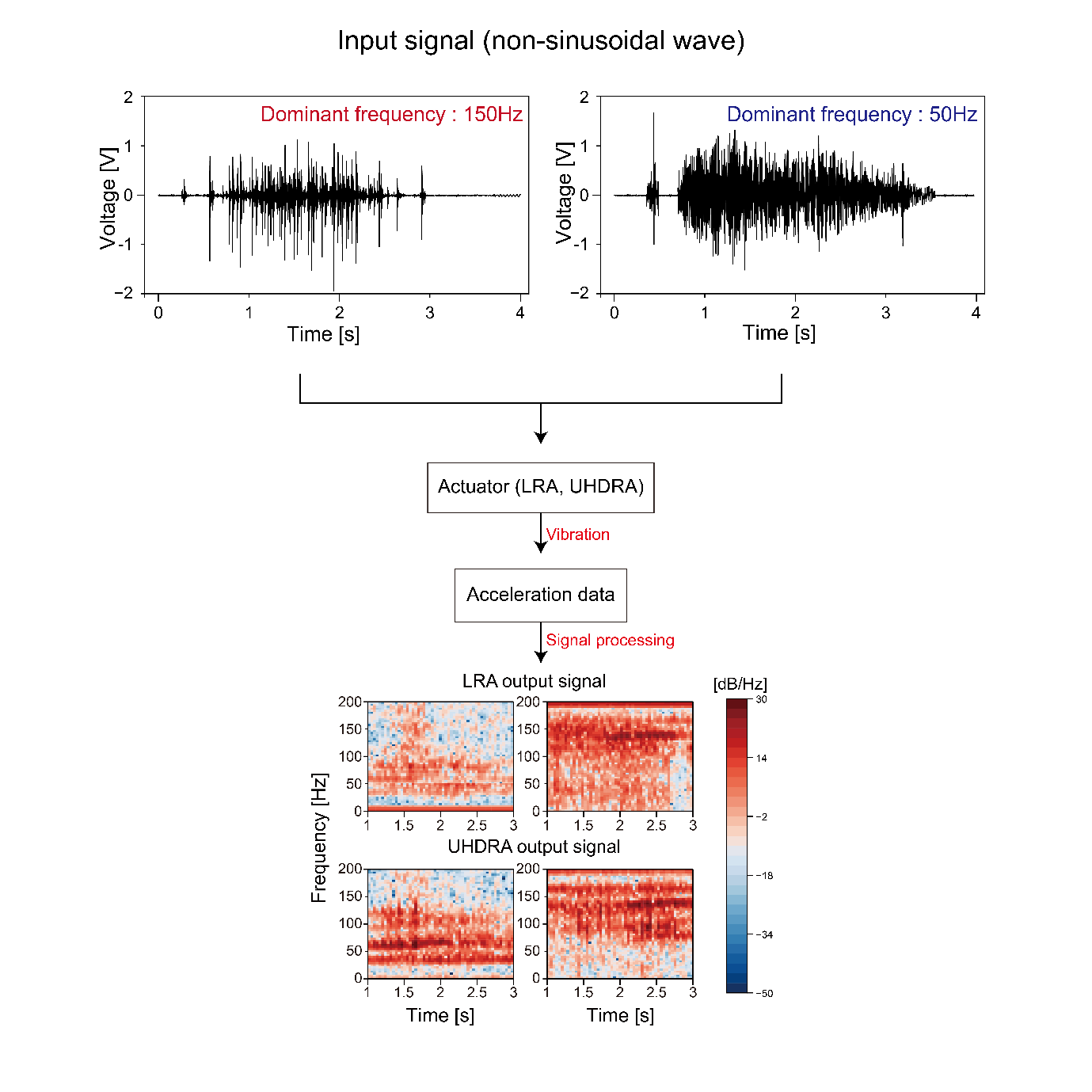


Figure S12. Spectrogram-based analysis of actuator operation under non-sinusoidal input vibrations. The acceleration signals obtained from the actuators were analyzed using spectrograms to clearly identify the dominant frequency components. These results demonstrate that both the LRA and UHDRA actuators can accurately reproduce and distinguish input vibrations with different dominant frequencies, validating their capability for frequency-selective haptic actuation.


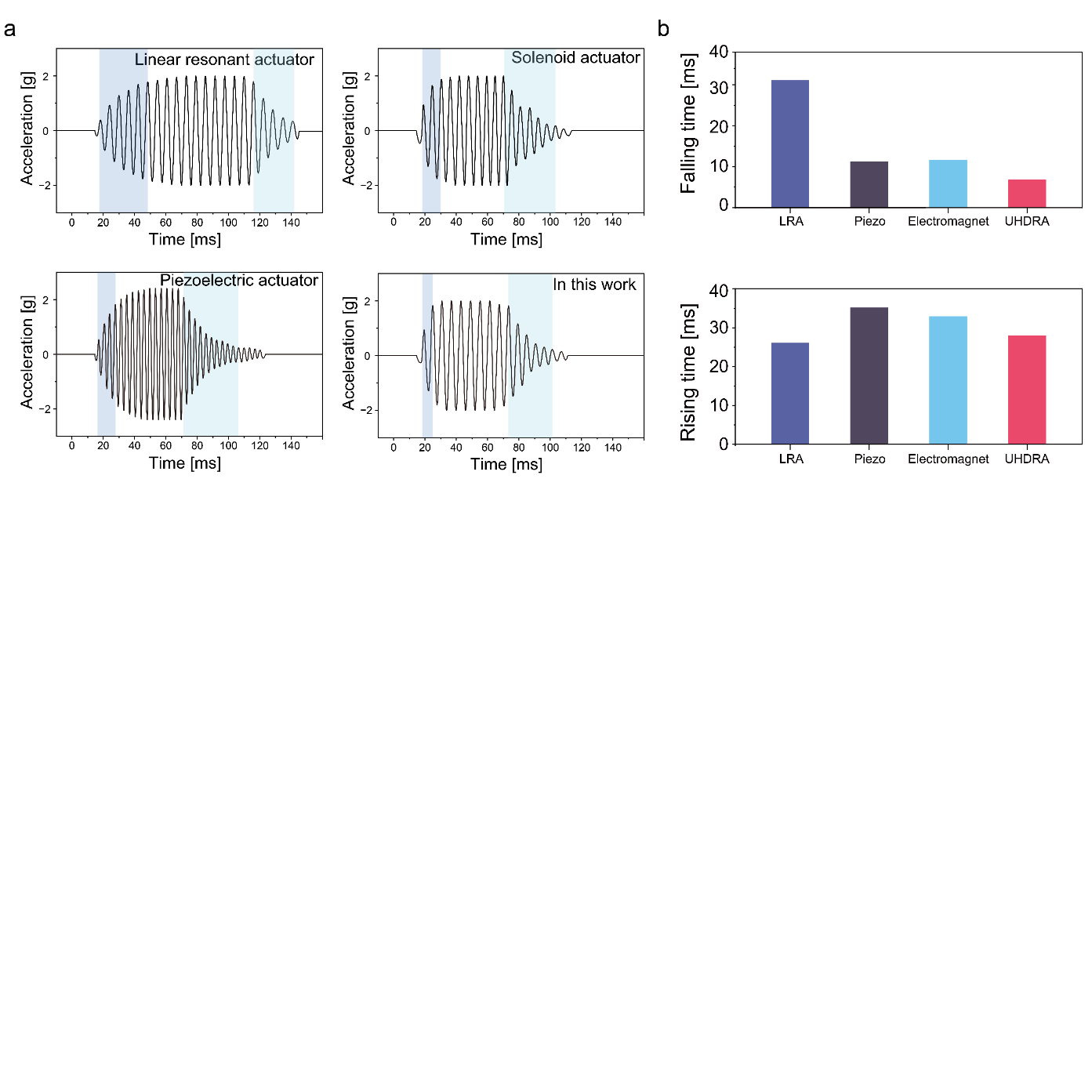


Figure S13. Response time comparison of different actuators. The comparison includes a linear resonant actuator (LRA), a piezoelectric actuator, an electromagnet actuator, and the proposed UHDRA. (a) Time-domain acceleration signals showing vibration decay after drive termination. (b) Extracted rising and falling times measured at the resonance point of each actuator.


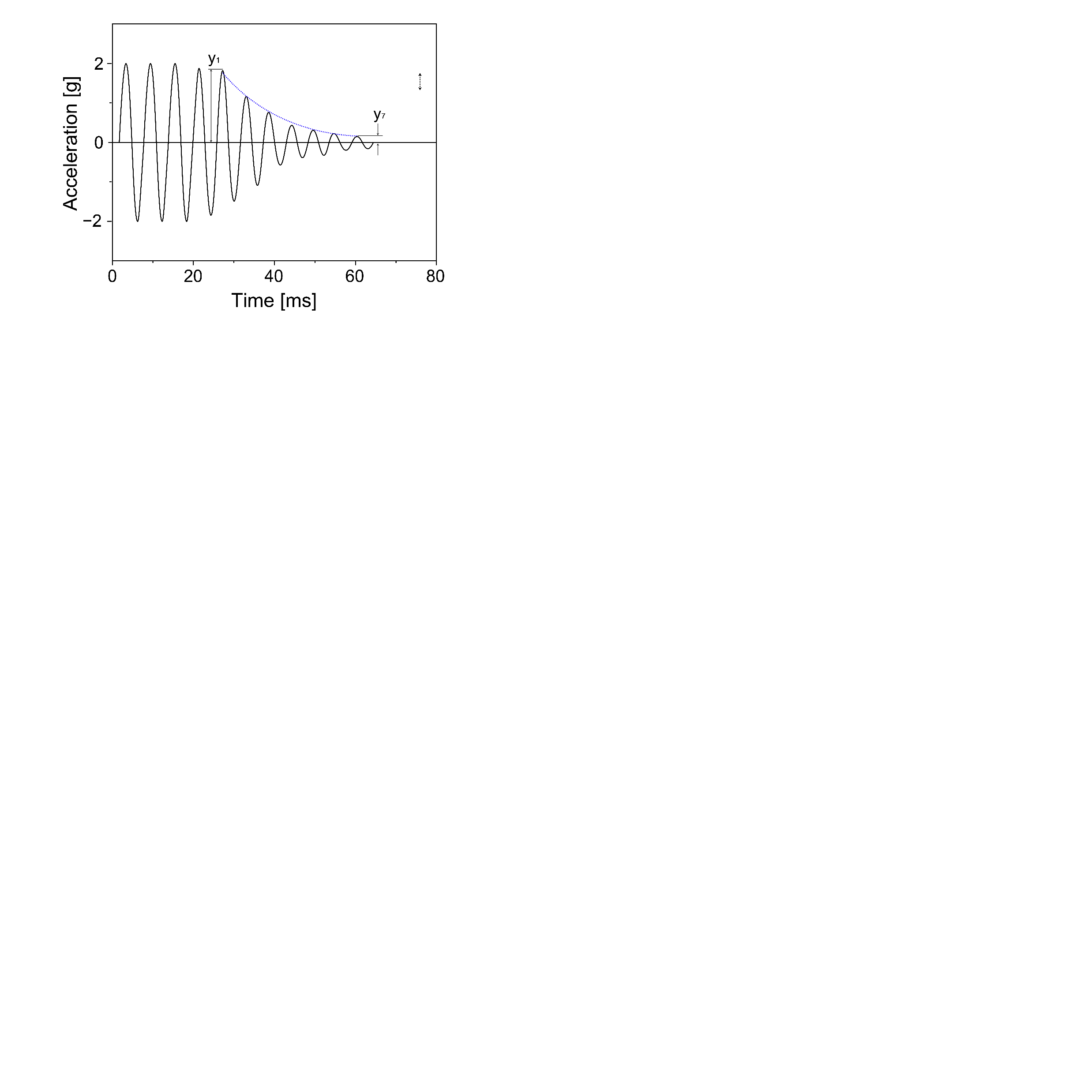


Figure S14. Calculation of damping ratio. Logarithmic decrement analysis of the free-decay vibration response, where the damping ratio is extracted from the exponential decay of successive peak amplitudes in the time-domain acceleration signal.


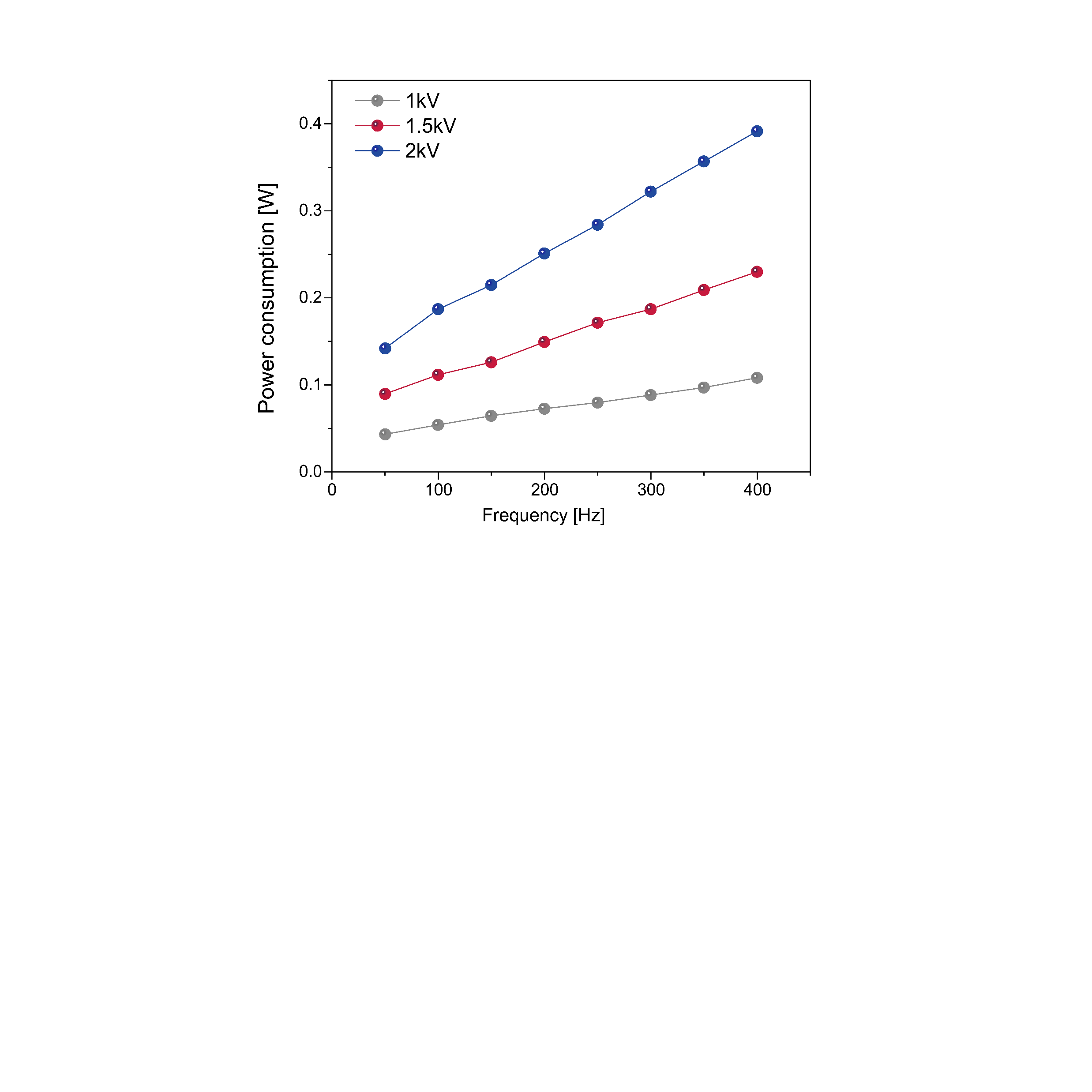


Figure S15. Power consumption of the UHDRA measured under different voltage input. The values represent the average power consumption obtained at both the low- and high-resonance modes of the actuator, shown as a function of frequency.


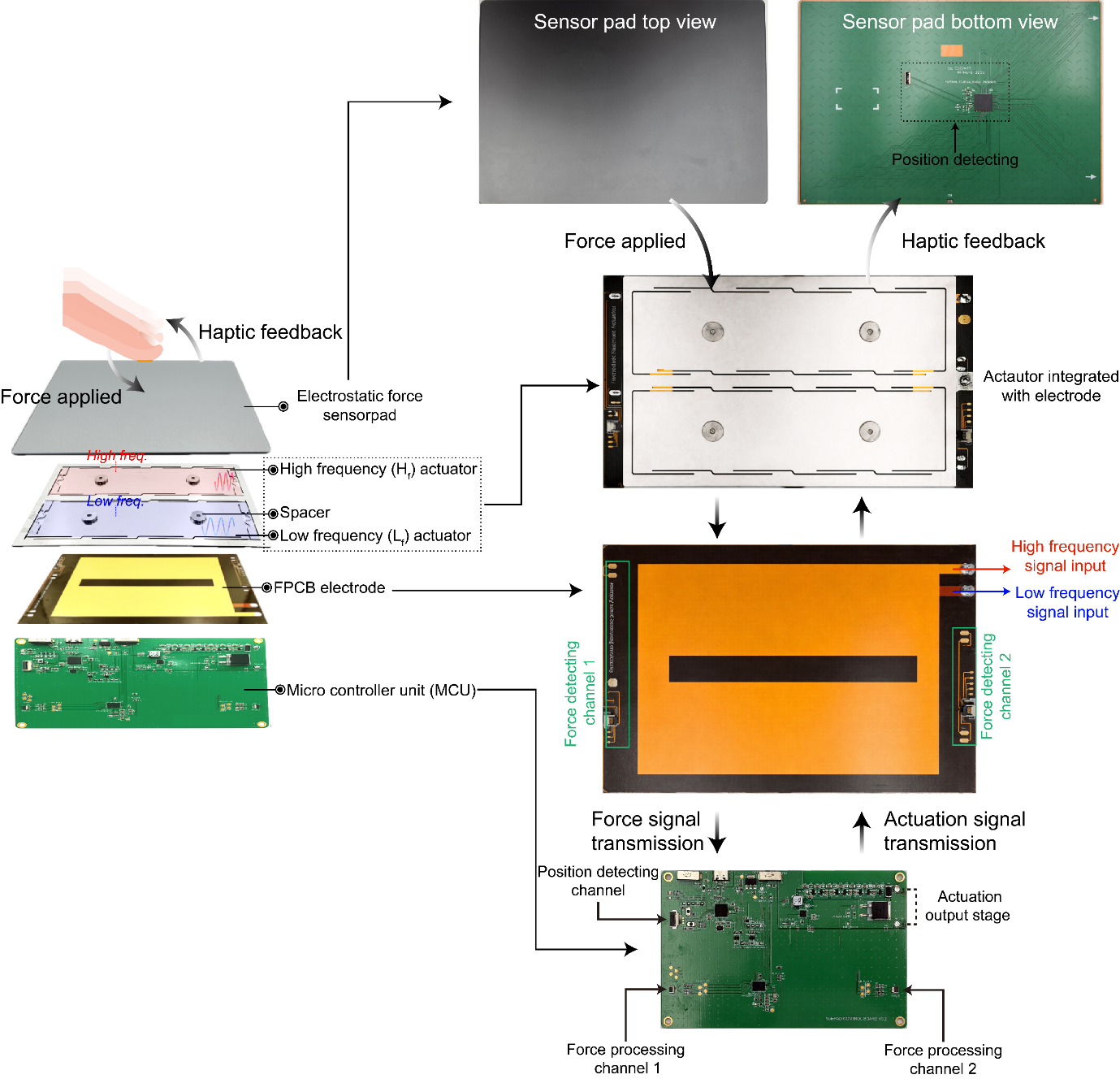


Figure S16. Expanded architecture of the integrated bidirectional HMI and its signal processing framework.

**
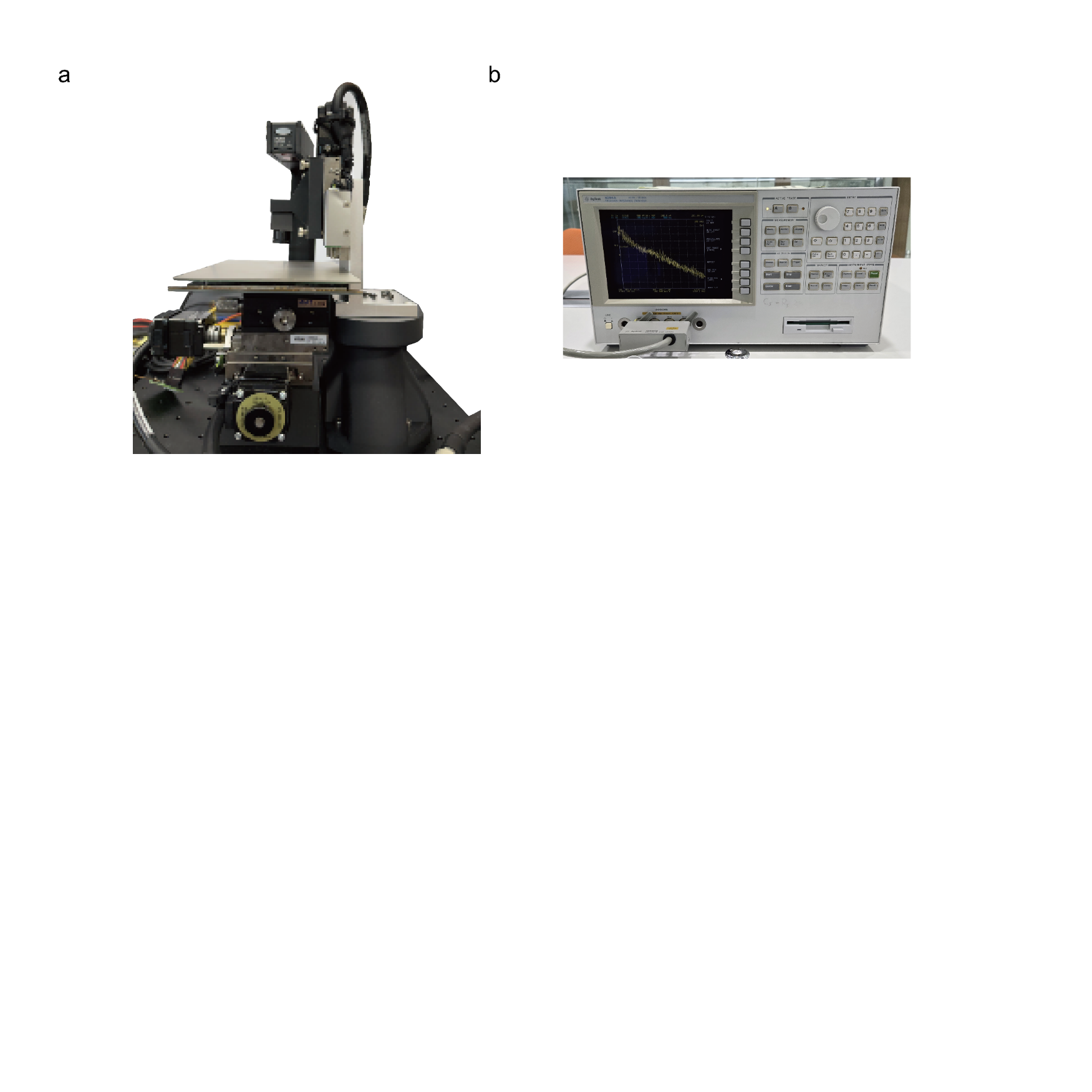
**

**Figure S17. Experimental setup for measuring capacitance variation of the UHDRA under applied contact force.** (a) Laptop touchpad integrated with the UHDRA positioned on a 3-axis force-controlled indentation system to apply controlled normal loads. (b) Impedance analyzer (Agilent 4294A) used to record the capacitance response of the UHDRA under varying contact forces.


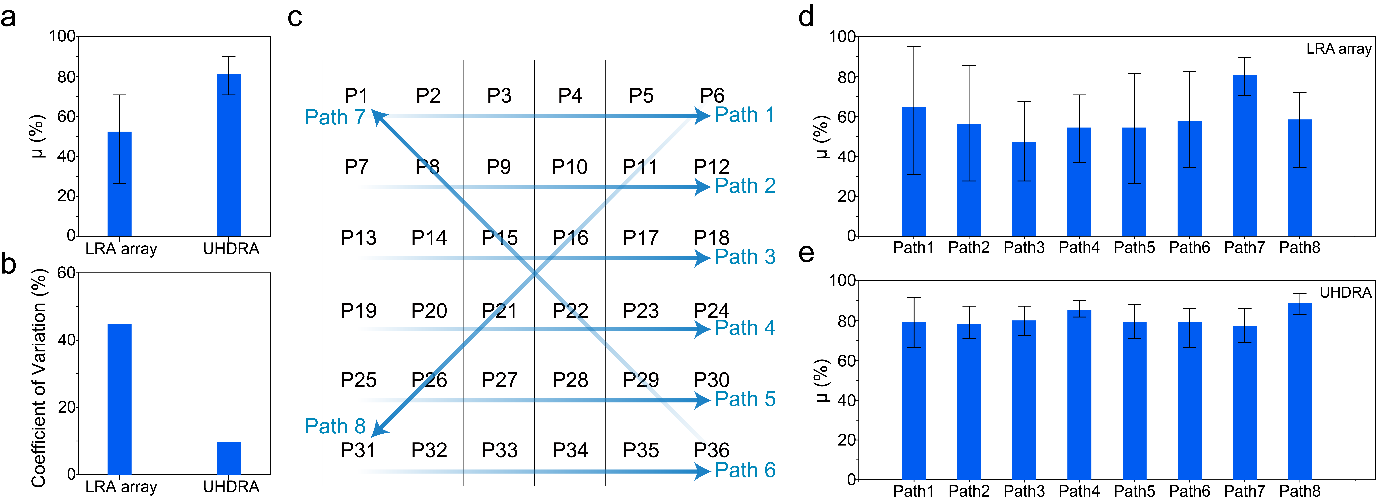


**Figure S18. Quantitative analysis of spatial vibration uniformity for the LRA array and UHDRA.** (a) Comparison of the mean vibration acceleration (μ) measured over the 36 sensing regions for the LRA array and UHDRA. (b) Coefficient of variation (CV) comparison, indicating the relative dispersion of vibration intensity across the sensing area. (c) Schematic of the 6 × 6 sensing grid used for uniformity evaluation, showing the eight analysis paths consisting of six horizontal paths (Path 1-6) and two diagonal paths (Path 7-8). (d) Mean vibration acceleration and standard deviation measured along each path for the LRA array. (e) Mean vibration acceleration and standard deviation measured along each path for the UHDRA, demonstrating improved spatial uniformity compared with the LRA array.


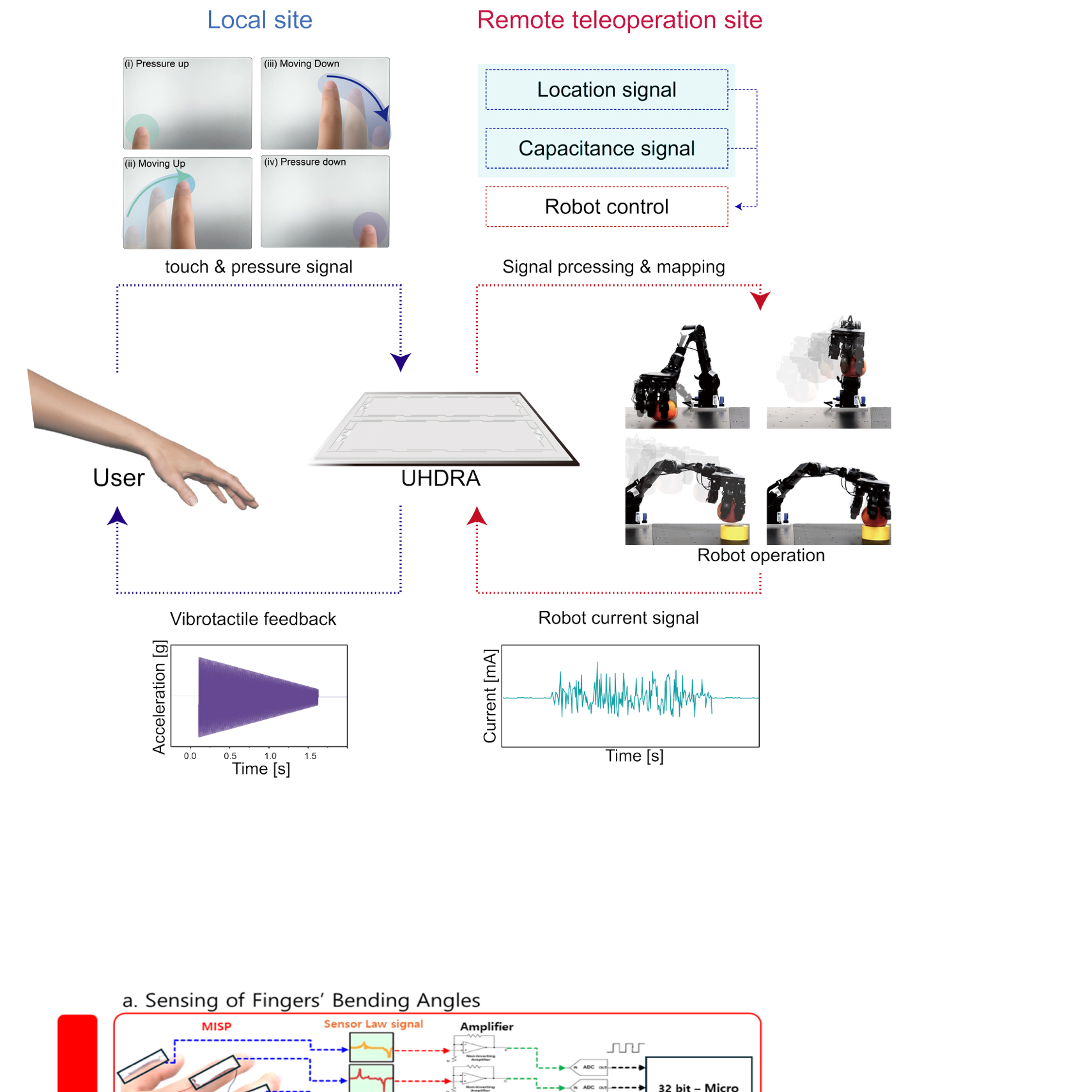


Figure S19. Bidirectional interaction between the user, actuator, and robot hand. Touch and pressure inputs from the user are detected by the UHDRA-integrated haptic interface and converted into capacitance signals that are processed for robot hand control. The resulting actuation commands drive the robotic hand, while feedback signals (current) from the robot are delivered back to the actuator to generate vibration feedback for the user.


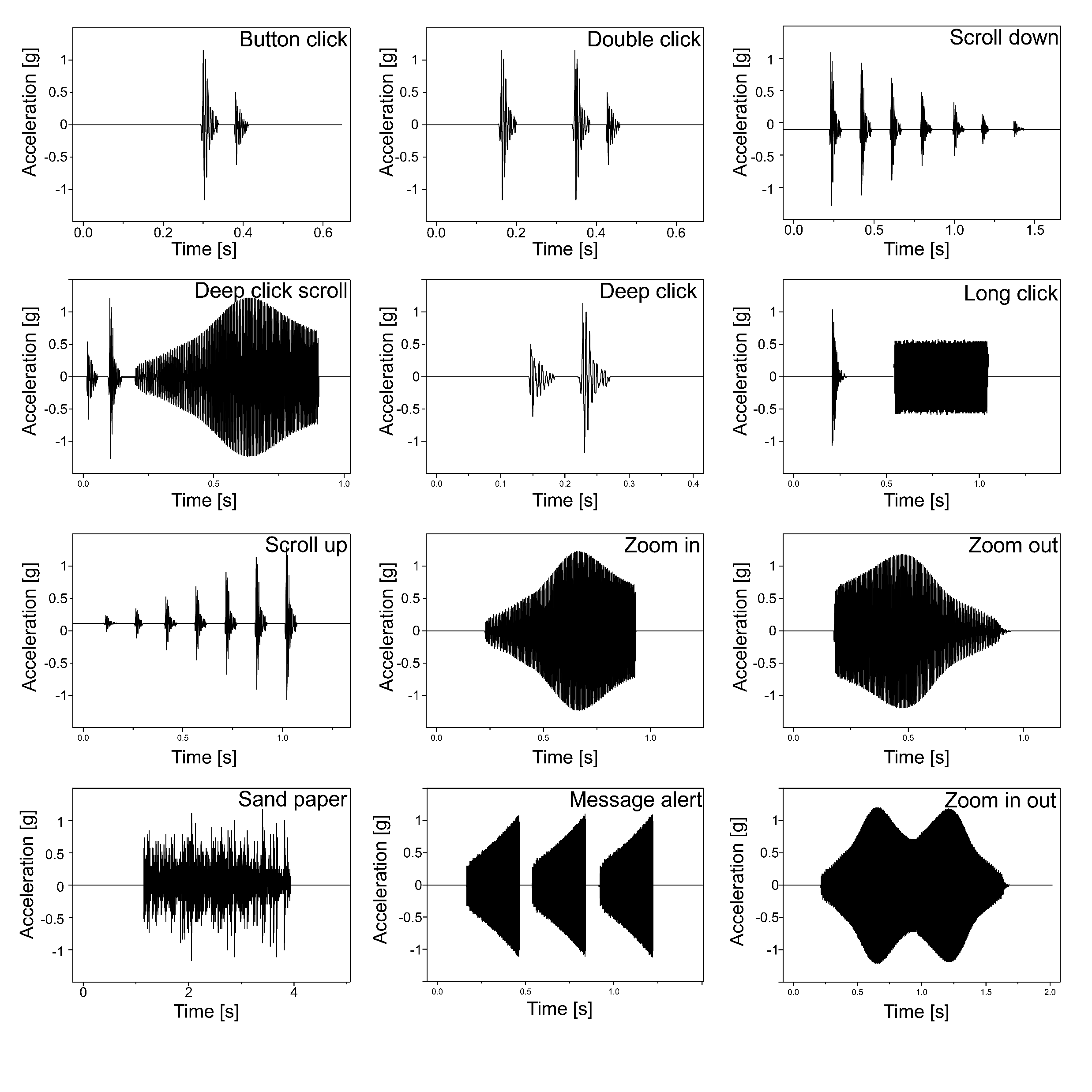


Figure S20. Vibration waveforms of the UHDRA for various interaction events and tactile feedback patterns, indicating dynamic modulation of feedback amplitude. Representative examples such as button clicks, scrolling actions, and zoom gestures are shown, highlighting the actuator’s capability to generate distinct waveforms tailored to different user interactions.


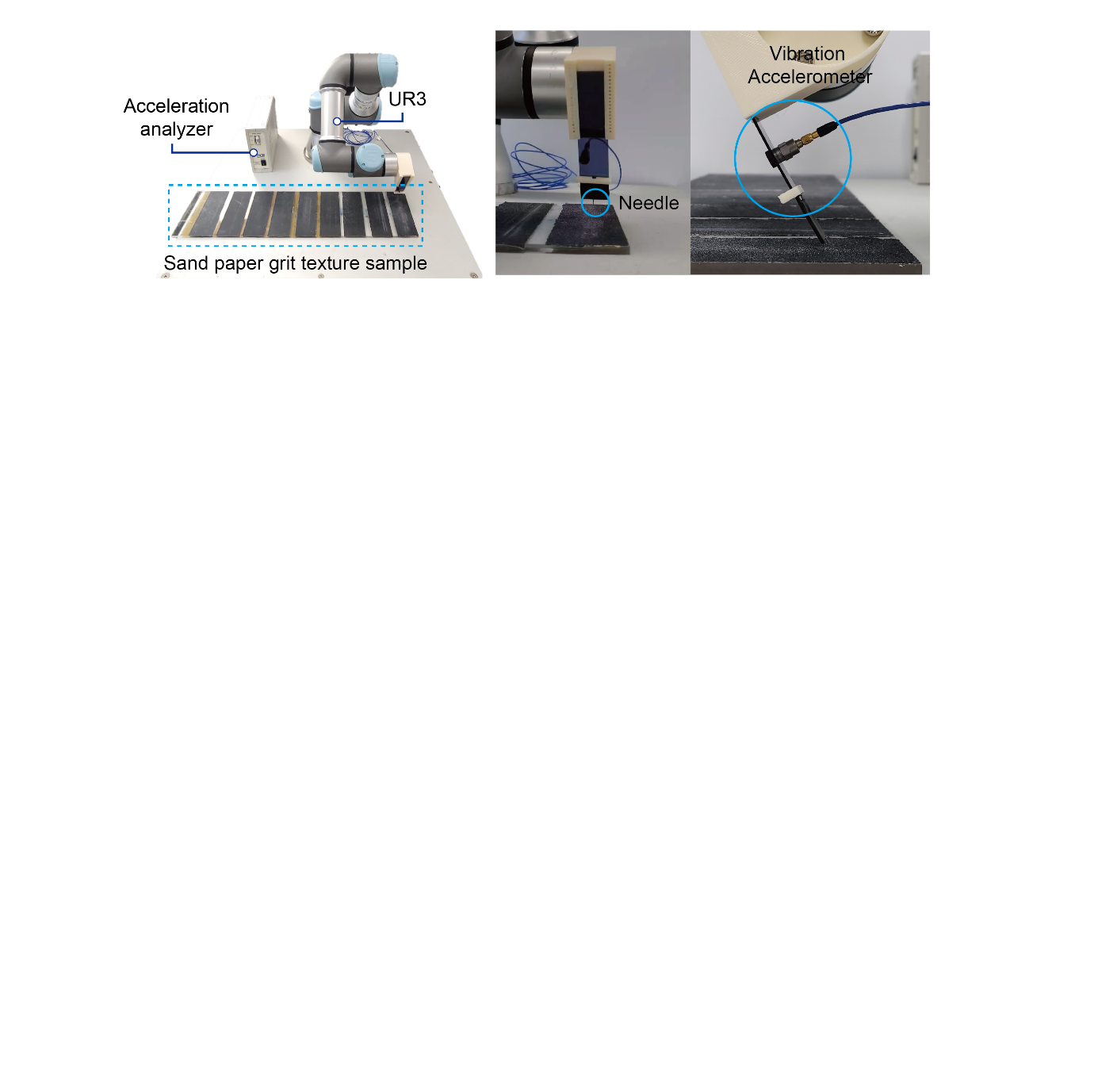


Figure S21. Experimental setup for vibration measurement using sandpaper grit texture samples. A UR3 robotic arm equipped with a needle probe and a vibration accelerometer records acceleration signal as the probe scans across surfaces with varying roughness. The acquired data are stored as DAQ files and subsequently analyzed to establish correlations between surface texture and vibration response, enabling quantitative evaluation of tactile texture representation.


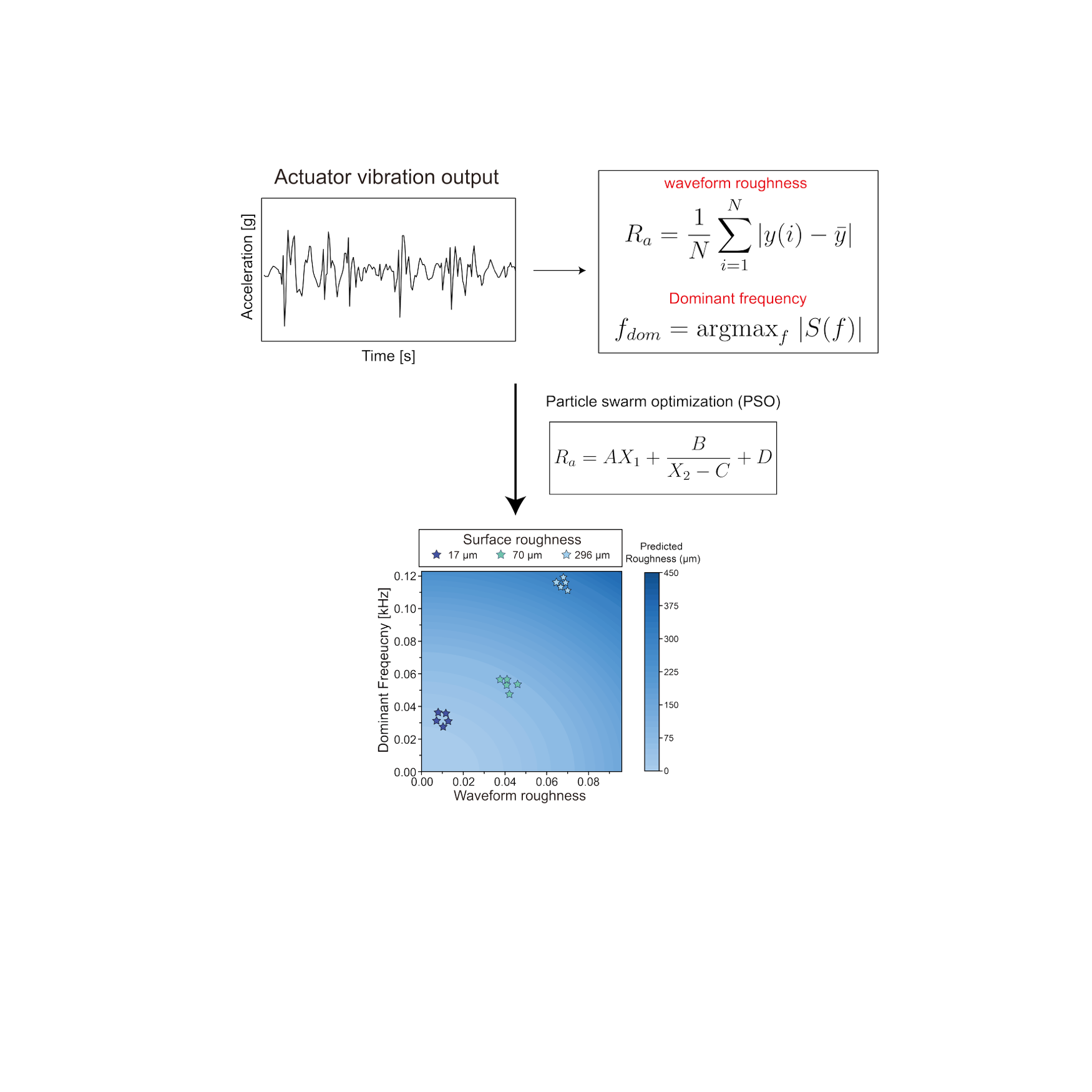


Figure S22. Prediction of surface roughness from actuator vibration signals. Waveform roughness (*R_a_*) and dominant frequency (*f_dom_*) were extracted from the vibration output and applied to a particle swarm optimization (PSO) model to estimate surface roughness. The resulting contour map illustrates predicted roughness values, with experimental validation points at 32 μm, 300 μm, and 632 μm.


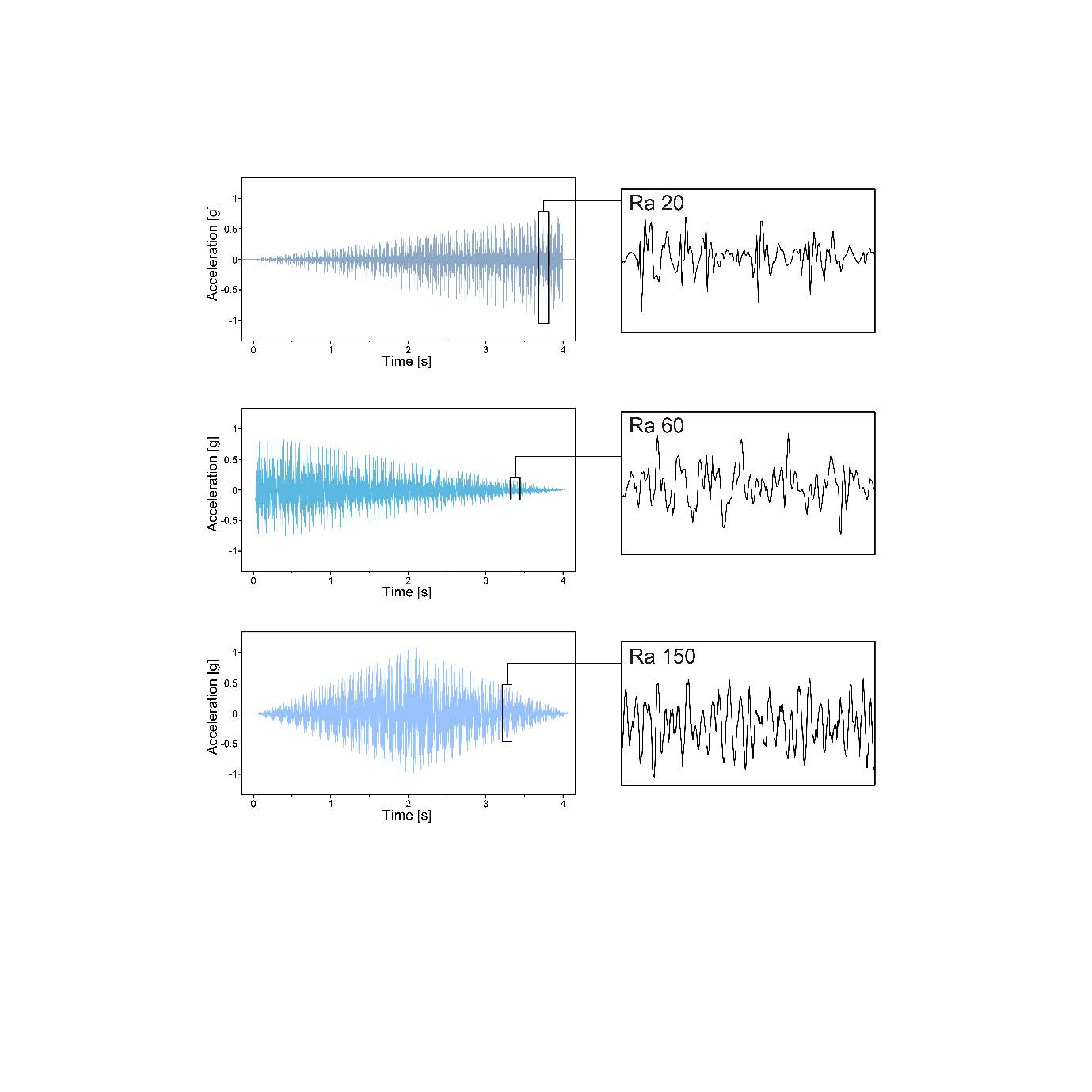


Figure S23. Representation of diverse tactile sensations by modulating vibration signals obtained from sandpapers with different roughness levels. Time-domain acceleration signals were amplified or attenuated to mimic variations in tactile intensity.

**References**

[1] Y.H. Heo, T.-H. Kim, D.-S. Choi, S.-Y. Kim, Head-up haptic display using dual-mode haptic actuator for vehicles, IEEE Transactions on Industrial Electronics, 71 (2023) 2779-2788.

[2] R. Hassanian, M. Riedel, N. Yeganeh, Numerical Investigation on the Acceleration Vibration Response of Linear Actuator, (2022).

[3] M. Hendijanizadeh, S. Sharkh, P. Mosca, S. Daley, An actuator with tuneable resonant frequency for active vibration damping, Journal of Intelligent Material Systems and Structures, 34 (2023) 1239-1252.

[4] W. McMahan, K.J. Kuchenbecker, Dynamic modeling and control of voice-coil actuators for high-fidelity display of haptic vibrations, 2014 IEEE Haptics Symposium (HAPTICS), IEEE, 2014, pp. 115-122.

[5] H.-Y. Yao, V. Hayward, Design and analysis of a recoil-type vibrotactile transducer, The Journal of the Acoustical Society of America, 128 (2010) 619-627.

[6] J. Nam, Y. Kim, G. Jang, Resonant piezoelectric vibrator with high displacement at haptic frequency for smart devices, IEEE/ASME Transactions on Mechatronics, 21 (2015) 394-401.

[7] J. Nam, H. Oh, G. Jang, Externally leveraged resonant piezoelectric actuator with fast response time for smart devices, IEEE/ASME Transactions on Mechatronics, 21 (2016) 2764-2772.

[8] S.-H. Liu, Y.-C. Kao, G.-H. Feng, Linear resonator actuator-constructed wearable haptic system with the application of converting remote grinding force to vibratory sensation, Actuators, MDPI, 2023, pp. 359.

[9] D. Pyo, T.-H. Yang, S. Ryu, D.-S. Kwon, Novel linear impact-resonant actuator for mobile applications, Sensors and Actuators A: Physical, 233 (2015) 460-471.

[10] J. Kim, G.W. Hwang, M. Song, D. Lim, J.I. Kim, J.H. Choi, Y.S. Lee, D.W. Kim, T.H. Yang, C. Pang, A reversible, versatile skin‐attached haptic interface platform with bioinspired interconnection architectures capable of resisting sweat and vibration, Advanced Functional Materials, 34 (2024) 2311167.

[11] M.T. Flavin, K.-H. Ha, Z. Guo, S. Li, J.-T. Kim, T. Saxena, D. Simatos, F. Al-Najjar, Y. Mao, S. Bandapalli, Bioelastic state recovery for haptic sensory substitution, Nature, 635 (2024) 345-352.

[12] J.I. Lindsay, I. Jiang, E. Larson, R. Adams, S.N. Patel, B. Hannaford, Good vibrations: An evaluation of vibrotactile impedance matching for low power wearable applications, Proceedings of the 26th annual ACM symposium on User interface software and technology, 2013, pp. 515-520.

[13] A. Dementyev, A. Olwal, R.F. Lyon, Haptics with input: back-EMF in linear resonant actuators to enable touch, pressure and environmental awareness, Proceedings of the 33rd annual ACM symposium on user interface software and technology, 2020, pp. 420-429.

[14] J.-T. Kim, H.-S. Shin, J.-Y. Yoo, R. Avila, Y. Huang, Y.H. Jung, J.E. Colgate, J.A. Rogers, Mechanics of vibrotactile sensors for applications in skin-interfaced haptic systems, Extreme Mechanics Letters, 58 (2023) 101940.

[15] N. Kastor, B. Dandu, V. Bassari, G. Reardon, Y. Visell, Ferrofluid electromagnetic actuators for high-fidelity haptic feedback, Sensors and Actuators A: Physical, 355 (2023) 114252.

[16] G. Yun, S. Choi, Real-time Semantic Full-Body Haptic Feedback Converted from Sound for Virtual Reality Gameplay, Proceedings of the 2025 CHI Conference on Human Factors in Computing Systems, 2025, pp. 1-17.
